# The loci of Stroop effects: a critical review of methods and evidence for levels of processing contributing to color-word Stroop effects and the implications for the loci of attentional selection

**DOI:** 10.1007/s00426-021-01554-x

**Published:** 2021-08-13

**Authors:** Benjamin A. Parris, Nabil Hasshim, Michael Wadsley, Maria Augustinova, Ludovic Ferrand

**Affiliations:** 1 Department of Psychology, Faculty of Science and Technology, Bournemouth University, Talbot Campus, Poole, Fern Barrow, BH12 5BB UK; 2 School of Psychology, University College Dublin, Dublin, Ireland; 3 Normandie Université, UNIROUEN, CRFDP, 76000 Rouen, France; 4 Université Clermont Auvergne, CNRS, LAPSCO, 63000 Clermont-Ferrand, France; 5 School of Applied Social Sciences, De Montfort University, Leicester, UK

## Abstract

Despite instructions to ignore the irrelevant word in the Stroop task, it robustly influences the time it takes to identify the color, leading to performance decrements (interference) or enhancements (facilitation). The present review addresses two questions: (1) What levels of processing contribute to Stroop effects; and (2) Where does attentional selection occur? The methods that are used in the Stroop literature to measure the candidate varieties of interference and facilitation are critically evaluated and the processing levels that contribute to Stroop effects are discussed. It is concluded that the literature does not provide clear evidence for a distinction between conflicting and facilitating representations at phonological, semantic and response levels (together referred to as informational conflict), because the methods do not currently permit their isolated measurement. In contrast, it is argued that the evidence for task conflict as being distinct from informational conflict is strong and, thus, that there are at least two loci of attentional selection in the Stroop task. Evidence suggests that task conflict occurs earlier, has a different developmental trajectory and is independently controlled which supports the notion of a separate mechanism of attentional selection. The modifying effects of response modes and evidence for Stroop effects at the level of response execution are also discussed. It is argued that multiple studies claiming to have distinguished response and semantic conflict have not done so unambiguously and that models of Stroop task performance need to be modified to more effectively account for the loci of Stroop effects.

## Introduction

In his doctoral dissertation, John R. Stroop was interested in the extent to which difficulties that accompany learning, such as interference, can be reduced by practice (Stroop, 1935). For this purpose, he construed a particular type of stimulus. Stroop displayed words in a color that was different from the one that they actually designated (e.g., the word *red* in blue font). After he failed to observe any interference from the colors on the time it took to read the words (Exp.1), he asked his participants to identify their font color. Because the meaning of these words (e.g., red) interfered with the to-be-named target color (e.g., blue), Stroop observed that naming aloud the color of these words takes longer than naming aloud the color of small squares included in his control condition (Exp.2). In line with both his expectations and other learning experiments carried out at the time, this interference decreased substantially over the course of practice. However, daily practice did not eliminate it completely (Exp.3). During the next thirty years, this result and more generally this paradigm received only modest interest from the scientific community (see, e.g., Jensen & Rohwer, 1966, MacLeod, 1992 for discussions). Things changed dramatically when color-word stimuli, ingeniously construed by Stroop, became a prime paradigm to study attention, and in particular selective attention (Klein, 1964).

The ability to selectively attend to and process only certain features in the environment while ignoring others is crucial in many everyday activities (e.g., Jackson & Balota, 2013). Indeed, it is this very ability that allows us to drive without being distracted by beautiful surroundings or to quickly find a friend in a hallway full of people. It is clear then that an ability to reduce the impact of potentially interfering information by selectively attending to the parts of the world that are consistent with our goals, is essential to functioning in the world as a purposive individual. The Stroop task (Stroop, 1935), as this paradigm is now known, is a selective attention task in that it requires participants to focus on one dimension of the stimulus whilst ignoring another dimension of the very same stimulus. When the word dimension is not successfully ignored, it elicits interference: Naming aloud the color that a word is printed in takes longer when the word denotes a different color (incongruent trials, e.g., the word *red* displayed in color-incongruent blue font) compared to a baseline condition. This difference in color-naming times is often referred to as *the* *Stroop* *interference* *effect* or *the* *Stroop* *effect* (see the section ‘Definitional issues’ for further development and clarifications of these terms).

Evidencing its utility, the Stroop task has been widely used in clinical settings as an aid to assess disorders related to frontal lobe and executive attention impairments (e.g., in attention deficit hyperactivity disorder, Barkley, 1997; schizophrenia, Henik & Salo, 2004; dementia, Spieler et al., 1996; and anxiety, Mathews & MacLeod, 1985; see MacLeod, 1991 for an in-depth review of the Stroop task). The Stroop task is also ubiquitously used in basic and applied research—as indicated by the fact that the original paper (Stroop, 1935) is one of the most cited in the history of psychology and cognitive science (e.g., Gazzaniga et al., 2013; MacLeod, 1992). It is, however, important to understand that the Stroop task as it is currently employed in neuropsychological practice (e.g., Strauss et al., 2007), its implementations in most basic and applied research (see here below), and leading accounts of the effect it produces, are profoundly rooted in the idea that the Stroop effect is a unitary phenomenon in that it is caused by the failure of a single mechanism (i.e., it has a single locus). By addressing the critical issue of whether there is a single locus or multiple loci of Stroop effects, the present review not only addresses several pending issues of theoretical and empirical importance, but also critically evaluates these current practices.

## The where vs. the when and the how of attentional control

The Stroop effect has been described as the gold standard measure of selective attention (MacLeod, 1992) in which a smaller Stroop interference effect is an indication of greater attentional selectivity. However, the notion that it is selective attention that is the cognitive mechanism enabling successful performance in the Stroop task has recently been sidelined (see Algom & Chajut, 2019, for a discussion of this issue). For example, in a recent description of the Stroop task, Braem et al. (2019) noted that the size of the Stroop congruency effect is “indicative of the signal strength of the irrelevant dimension relative to the relevant dimension, as well as of the level of *cognitive* *control* applied” (p769). Cognitive control is a broader concept than selective attention in that it refers to the entirety of mechanisms used to control thought and behavior to ensure goal-oriented behavior (e.g., task switching, response inhibition, working memory). Its invocation in describing the Stroop task has proven to be somewhat controversial given that it implies the operation of top-down mechanisms, which might or might not be necessary to explain certain experimental findings (Algom & Chajut, 2019; Braem et al., 2019; Schmidt, 2018). It does, however, have the benefit of hypothesizing a form of attentional control that is not a static, invariant process but instead posits a more dynamic, adaptive form of attentional control, and provides foundational hypotheses about how and when attentional control might happen. However, the present work addresses that which the cognitive control approach tends to eschew (see Algom & Chajut, 2019): the question of where the conflict that causes the interference comes from. Importantly, the answer to the where question will have implication for the how and when questions.

The question of where the interference derives has historically been referred to as the locus of the Stroop effect (e.g., Dyer, 1973; Logan & Zbrodoff, 1998, Luo, 1999; Scheibe et al., 1967; Seymour, 1977; Wheeler, 1977; see also MacLeod, 1991, and Parris, Augustinova & Ferrand, 2019). Whilst, by virtue of our interest in where attentional selection occurs, we review evidence for the early or late selection of information in the color-word Stroop task, recent models of selective attention have shown that whether selection is early or late is a function of either the attentional resources available to process the irrelevant stimulus (Lavie, 1995) or the strength of the perceptual representation of the irrelevant dimension (Tsal & Benoni, 2010). Moreover, despite being referred to as the gold standard attentional measure and as one of the most robust findings in the field of psychology (MacLeod, 1992), it is clear that Stroop effects can be substantially reduced or eliminated by making what appear to be small changes to the task. For example, Besner, Stolz, and Boutillier (1997) showed that the Stroop effect can be reduced and even eliminated by coloring a single letter instead of all letters of the irrelevant word (although notably they used button press responses which produced smaller Stroop effects (Sharma & McKenna, 1998) making it easier to eliminate interference; see also Parris, Sharma, & Weekes, 2007). In addition, Melara and Mounts (1993) showed that by making the irrelevant words smaller to equate the discriminability of word and color, the Stroop effect can be eliminated and even reversed.

Later, Dishon-Berkovits and Algom (2000) noted that often in the Stroop task the dimensions are correlated in that one dimension can be used to predict the other (i.e., when an experimenter matches the number of congruent (e.g., the word *red* presented in the color red) and incongruent trials in the Stroop task, the irrelevant word is more often presented in its matching color than in any other color which sets up a response contingency). They demonstrated that when this dimensional correlation was removed the Stroop effect was substantially reduced. By showing that the Stroop effect is malleable through the modulation of dimensional uncertainty (degree of correlation of the dimensional values and how expected the co-occurrences are) or dimensional imbalance (of the salience of each dimension) their data, and resulting model (Melara & Algom, 2003; see also Algom & Fitousi, 2016), indicate that selective attention is failing because the experimental set-up of the Stroop task provides a context with little or no perceptual load / little or no perceptual competition, and where the dimensions (word and color) are often correlated and / or asymmetrical in discriminability that contributes to the robust nature of the Stroop effect. In other words, the Stroop task sets selective attention mechanisms up to fail, pitching as it does the intention to ignore irrelevant information against the tendency and resources to process conspicuous and correlated characteristics of the environment (Melara & Algom, 2003). But, in the same way that neuropsychological impairments teach us something about how the mind works (Shallice, 1988), it is these failures that give us an opportunity to explore the architecture of the mechanisms of selective attention in healthy and impaired populations. We, therefore, ask the question: if control does fail, where (at what levels of processing) is conflict experienced in the color-word Stroop task?

Given our focus on the varieties of conflict (and facilitation), the *where* of control, we will not concern ourselves with the how and the when of control. Manipulations and models of the Stroop task that are not designed to understand the types of conflict and facilitation that contribute to Stroop effects such as list-wise versus item-specific congruency proportion manipulations (e.g., Botvinick et al., 2001; Bugg, & Crump, 2012; Gonthier et al., 2016; Logan & Zbrodoff, 1979; Schmidt & Besner, 2008; Schmidt, Notebaert, & Van Den Bussche, 2015; see Schmidt, 2019, for a review) or memory load manipulations (e.g., De Fockert, 2013; Kalanthroff et al., 2015; Kim et al., 2005; Kim, Min, Kim & Won, 2006), will be eschewed, unless these manipulations are specifically modified in a way that permits the understanding of the processing involved in producing Stroop interference and facilitation. To reiterate the aims of the present review, here we are less concerned with the evaluative function of control which judges when and how control operates (Chuderski & Smolen, 2016), but are instead concerned with the regulative function of control and specifically at which processing levels this might occur. In short, the present review attempts to identify whether at any level, other than the historically favoured level of response output, processing reliably leads to conflict (or facilitation) between activated representations. Before we address this question, however, we must first address the terminology used here and, in the literature, to describe different types of Stroop effects.

## Definitional issues to consider before we begin

### A word about baselines and descriptions of Stroop effects

**Fig. 1:**
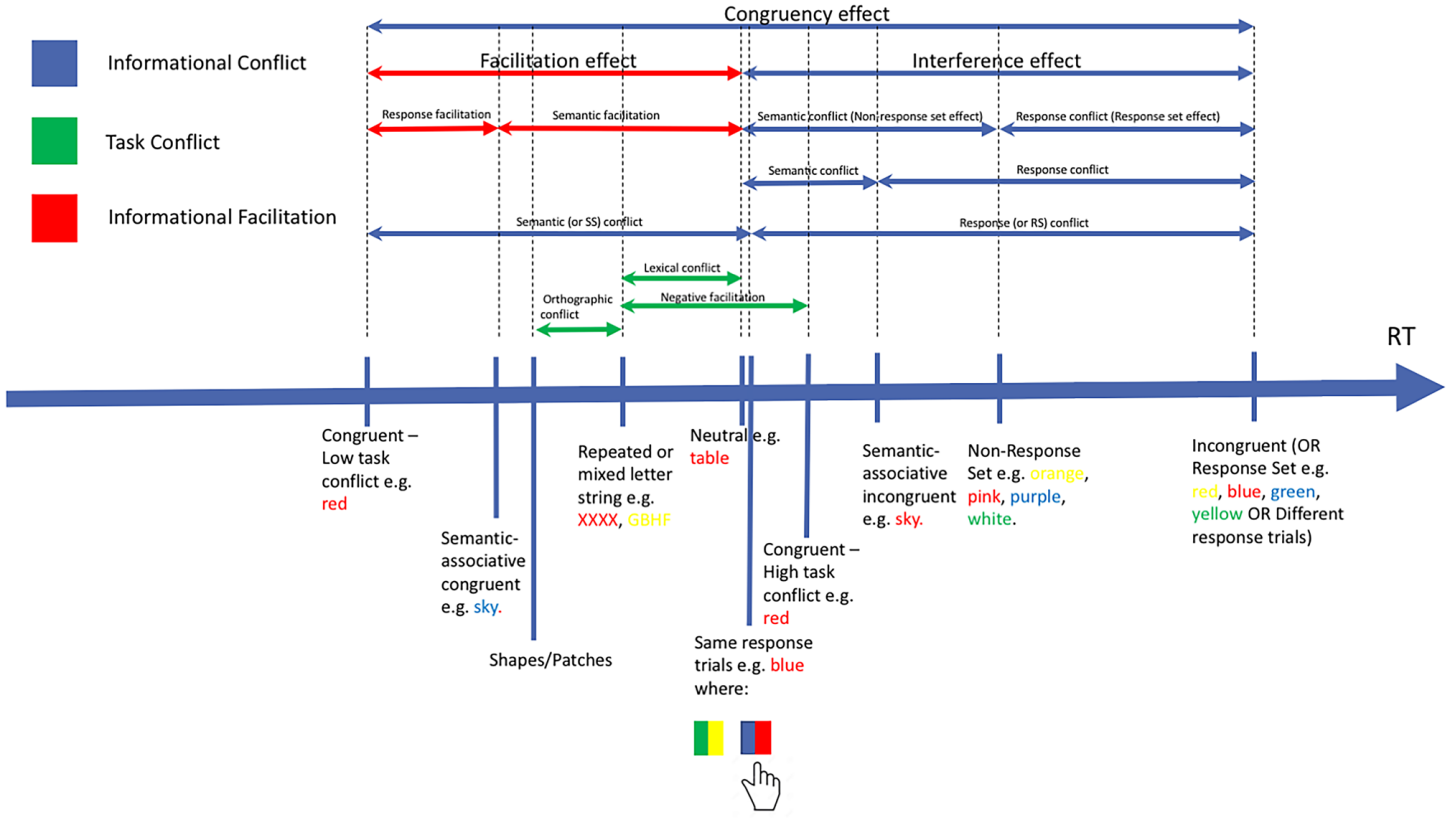
This figure shows examples of the various trial types that have been used to decompose the Stroop effect into various types of conflict (interference) and facilitation. This has resulted in a lack of clarity about what components are being measured. Indeed, as can be seen, one person’s semantic conflict can be another person’s facilitation, a problem that arises due to the selection of the baseline control condition

Given the number of studies that have employed the Stroop task since its inception in 1935, it is no surprise that a variety of modifications of the original task have been employed, including the introduction of new trial types (as exemplified by Klein, 1964) and new ways of responding, to measure and understand mechanisms of selective attention. This has led to disagreement over what is being measured by each manipulation, obfuscating the path to theoretical enlightenment. Various trial types have been used to distinguish types of conflict and facilitation in the color-word Stroop task (see Fig. 1), although with less fervor for facilitation varieties, resulting in a lack of agreement about how one should go about indexing response conflict, semantic conflict, and other forms of conflict and facilitation. Indeed, as can be seen in Fig. 1, one person’s semantic conflict can be another person’s facilitation; a problem that arises due to the selection of the baseline control condition. Differences in performance between a critical trial and a control trial might be attributed to a specific variable but this method relies on having a suitable baseline that differs only in the specific component under test (Jonides & Mack, 1984).

Selecting an appropriate baseline, and indeed an appropriate critical trial, to measure the specific component under test is non-trivial. For example, congruent trials, first introduced by Dalrymple-Alford and Budayr (1966, Exp. 2), have become a popular baseline condition against which to compare performance on incongruent trials. Congruent trials are commonly responded to much faster than incongruent trials and the difference in reaction time between the two conditions has been variously referred to as the Stroop congruency effect (e.g., Egner et al., 2010), the Stroop interference effect (e.g., Leung et al., 2000), and the Total Stroop Effect (Brown et al., 1998), and Color-Word Impact (Kahneman & Chajczyk, 1983). However, when compared to non-color-word neutral trials, congruent trials are often reported to be responded to faster, evidencing a facilitation effect of the irrelevant word on the task of color naming (Dalrymple-Alford, 1972; Dalrymple-Alford & Budayr, 1966). Referring to the difference between incongruent and congruent trials as Stroop interference then—as is often the case in the Stroop literature—fails to recognize the role of facilitation observed on congruent trials and epitomizes a wider problem. As already emphasized by MacLeod (1991), this difference corresponds to “(…) the sum of facilitation and interference, each in unknown amounts” (MacLeod, 1991, p.168). Moreover, as will be discussed in detail later, congruent trial reaction times have been shown to be influenced by a newly discovered form of conflict, known as task conflict (Goldfarb & Henik, 2007) and are not, therefore, straightforwardly a measure of facilitation either.

Furthermore, whilst the common implementation of the Stroop task involves incongruent, congruent, and non-color-word neutral trials (or perhaps where the non-color-word neutral baseline is replaced by repeated letter strings e.g., xxxx), this common format ignores the possibility that the difference between incongruent and neutral trials involves multiple processes (e.g., semantic and response level conflict). As Klein (1964) showed the irrelevant word in the Stroop task can refer to concepts semantically associated with a color (e.g., sky; Klein, 1964), potentially permitting a way to answer to the question of whether selection occurs early at the level of semantics, before response selection, in the processing stream. But it is unclear whether such trials are direct measures of semantic conflict or indirect measures of response conflict.

Here, we employ the following terms: We refer to the difference between incongruent and congruent conditions as the *Stroop* *congruency* *effect*, because it contrasts performance in conditions with opposite congruency values. For the reasons noted above, the term *Stroop* *interference* or just *interference* is preferentially reserved for referring to slower performance on one trial type compared to another. The word *conflict* will denote competing representations at any particular level that could be the cause of interference (note that interference might not result from conflict (De Houwer, 2003) as, for example, in the emotional Stroop task, interference could result without conflict from competing representations (Algom et al., 2004)). When the distinction is not critical, the terms interference and conflict will be used interchangeably. The term *Stroop* *facilitation* or just *facilitation* will refer to the speeding up of performance on one trial type compared to another (unless specified otherwise). In common with the literature, facilitation will also be used to refer to the opposite of conflict; that is, it will denote facilitating representations at any level. Finally, the term *Stroop* *effect(s)* will be employed to refer more generally to all of these effects.

## Levels of conflict vs. levels of selection

When considering the standard incongruent Stroop trial (e.g., *red* in blue) where the word dimension is a color word (e.g., red) that is incongruent with the target color dimension that is being named, and where the color red is also a potential response, one might surmise numerous levels of representation where these two concepts might compete. Processing of the color dimension of a Stroop stimulus to name the color would, on a simple analysis, require initial visual processing, followed by activation of the relevant semantic representation and then word-form (phonetic) encoding of the color name in preparation for a response. For this process to advance unimpeded until response there would need to be no competing representations activated at any of those stages. Like color naming, the processes of word reading also requires visual processing but of letters and not of colors perhaps avoiding creating conflict at this level, although there is evidence for a competition for resources at the level of visual processing under some conditions (Kahneman & Chajczyk, 1983). Word reading also requires the computation of phonology from orthography which color processing does not. One way interference might occur at this level is if semantic processing or word-form encoding during the processing of the color dimension also leads to the unnecessary (for the purposes of providing a correct response) activation of the orthographic representation of the color name—as far as we are aware there is no evidence for this. However, orthography does appear to lead to conflict through a different route—the presence of a word or word-like stimulus appears to activate the full mental machinery used to process words. This unintentionally activated word reading task set, conflicts with the intentionally activated color identification task set, creating task conflict. Task conflict occurs whenever an orthographically plausible letter string is presented (e.g., the word *table* leads to interference, as does the non-word but pronounceable letter string *fanit*; the letter string *xxxxx* less so; Levin & Tzelgov, 2016; Monsell et al., 2001).

Despite being a task in which participants do not intend to engage, irrelevant word processing would also likely involve the activation of a phonological representation of the word and the activation of a semantic representation (and likely some word-form encoding), either of which could lead to the activation of representations competing for selection. However, just because the word is processed at certain level (e.g., orthography or phonology here) does not mean that each of these levels independently lead to conflict. Phonological information would only independently contribute to conflict if the process of color naming activated a competing representation at the same level. Otherwise, the phonological representation of the irrelevant word might simply facilitate activation of the semantic representation of the irrelevant word thereby providing competition for the semantic representation of the relevant color. In which case, whilst phonological information would contribute to Stroop effects, no selection mechanism would be required at the phonological level. And of course, there could be conflict at the phonological processing level, but with no selection mechanism available, conflict would have to be resolved later. To identify whether selection occurs at the level of phonological processing, a method would be needed to isolate phonological information from information at the semantic and response levels.

So-called late selection accounts would argue that any activated representations at these levels would result in increased activation at the response level where selection would occur with no competition or selection at earlier stages (e.g., Dyer, 1973; Logan & Zbrodoff, 1998, Luo, 1999; Scheibe et al., 1967; Seymour, 1977; Wheeler, 1977; see also MacLeod, 1991, and Parris, Augustinova & Ferrand, 2019a, 2019b, 2019c; for discussions of this topic). In contrast, so-called early selection accounts (De Houwer, 2003; Scheibe et al., 1967; Seymour, 1977; Stirling, 1979; Zhang & Kornblum, 1998; Zhang et al., 1999) argue for earlier and multiple sites of attentional selection with Hock and Egeth (1970) even arguing that the perceptual encoding of the color dimension is slowed by the irrelevant word, although this has been shown to be a problematic interpretation of their results (Dyer, 1973). In Zhang and colleagues models, attentional selection occurred and was resolved at the stimulus identification stage, before any information was passed on to the response level which had its own selection mechanism.

## The organization of the review

It is important to emphasize at this point then that when considering the locus or loci of the Stroop effect, there are in fact two issues to address. The first concerns the level(s) of processing that significantly contribute to Stroop interference (and facilitation) so that a specific type of conflict actually arises at this level. The second issue concerns the level(s) of attentional selection: Is there, like Zhang and Kornblum (1998) and Zhang et al. (1999) have suggested, more than one level at which attentional selection occurs?

With regards to the first issue, we start below by critically evaluating the evidence for different levels of processing that putatively contribute to conflict with the objective of assessing the methods used to index the forms of conflict, and what we can learn from them. To do this, we employed the distinction introduced by MacLeod and MacDonald (2000) who argued for two categories of conflict: *informational* and the aforementioned *task* conflict (see also Levin & Tzelgov, 2016) to further structure the review. Informational conflict arises from the *semantic* and *response* information that the irrelevant word conveys. This roughly corresponds to the distinction between stimulus-based and response-based conflicts (Kornblum & Lee, 1995; Kornblum et al., 1990; Zhang & Kornblum, 1998; Zhang et al., 1999). According to this approach, conflict arises due to overlap between the dimensions of the Stroop stimulus at the level of stimulus processing (Stimulus–Stimulus or S–S overlap) and at the level of response production (Stimulus–Response or S–R overlap). At the level of stimulus processing interference can occur at the perceptual encoding, memory retrieval, conceptual encoding and stimulus comparison stages. At the level of response production interference can also occur at response selection, motor programming and response execution. In the Stroop task, the relevant and irrelevant dimensions both involve colors and would, thus, produce Stimulus–Stimulus conflict and both stimuli overlap with the response (S–R overlap) because the response involves color classification. We also include phonological processing and word frequency in the informational conflict taxon (cf. Levin & Tzelgov, 2016). We discuss informational conflict and its varieties in the first section which is entitled ‘Decomposing Informational conflict’.

Task conflict, as noted above, arises when two task sets compete for resources. In the Stroop task, the task set for color identification is endogenously and purposively activated, and the task set for word reading is exogenously activated on presentation of the word. The simultaneous activation of two task sets creates conflict even before the identities of the Stroop dimensions have been processed. Therefore, this form of conflict is generated by all irrelevant words in the Stroop task including congruent and neutral words (Monsell et al., 2001). We discuss task conflict in the section ‘Task conflict’. We then discuss the often overlooked phenomenon of Stroop facilitation in the section entitled ‘Informational facilitation’. In the section entitled “Other evidence relevant to the issue of locus vs. loci of the Stroop effect” we consider the influence of response mode (vocal, manual, oculomotor) on the variety of conflicts and facilitation observed in the subsection ‘Response modes and the loci of the Stroop effect’ and we consider whether conflict and facilitation effects are resolved even once a response has been favored in the subsection ‘Beyond response selection: Stroop effects on response execution’. In the final section entitled “Locus or loci of selection?”, we use the outcome of these deliberations to discuss the second issue of whether the evidence supports attentional selection at a single or at multiple loci.

## Decomposing informational conflict

A seminal paper by George S. Klein in 1964 (Klein, 1964) represents a critical impetus for understanding different types of informational conflict. Indeed, up until Klein, all studies had utilized incongruent color-word stimuli as the irrelevant dimension. Klein was the first to manipulate the relatedness of the irrelevant word to the relevant color responses to determine the “evocative strength of the printed word” (1964, p. 577). To this end, he compared color-naming times of lists of nonsense syllables, low-frequency non-color-related words, high-frequency non-color words, words with color-related meanings (semantic associates: e.g., lemon, frog, sky), color words that were not in the set of possible response colors (non-response set stimuli), and color words that were in the set of possible response colors (response set stimuli). The response times increased linearly in the order they are presented above. Whilst lists of nonsense syllables vs. low-frequency words, high-frequency words vs. semantic-associative stimuli, and semantic-associative stimuli vs. non-response set stimuli did not differ, all other comparisons were significant.

It is important to underscore that for Klein himself, there was no competition between semantic nodes or at any stage of processing, and, thus, no need for attentional selection other than at the response stage. Only when both irrelevant word and relevant color are processed to the point of providing evidence towards different motor responses, do the two sources of information compete. Said differently, whilst he questioned the effect of semantic relatedness, Klein assumed that semantic relatedness would only affect the strength of activation of alternative motor responses. Highlighting his favoring of a single late locus for attentional selection, Klein noted that words that are semantically distant from the color name would be less likely to “arouse the associated motor-response in competitive intensity” (p. 577). Although others (e.g., early selection accounts mentioned above) have argued for competition and selection occurring earlier than response output, a historically favored view of the Stroop interference effect as resulting solely from response conflict has prevailed (MacLeod, 1991) such that so-called informational conflict (MacLeod & MacDonald, 2000) is viewed as being essentially solely response conflict. That is, the color and word dimensions are processed sufficiently to produce evidence towards different responses and before the word dimension is incorrectly selected, mechanisms of selective attention at response output have to either inhibit the incorrect response or bias the correct response.

### Response and semantic level processing

To assess the extent to which we can (or cannot) move forward from this latter view, we describe and critically evaluate methods used to dissociate and measure the potentially independent contributions of response and semantic conflict. We start by considering so-called *same-response* trials before going on to consider semantic-associative trials, non-response set trials and a method that has used semantic distance on the electromagnetic spectrum as a way to determine the involvement of semantic conflict in the color-word Stroop task. Indeed, this is an important first step for determining whether at this point informational conflict can (or cannot) be reliably decomposed.

### Same-response trials

Same-response trials utilize a two-to-one color-response mapping and have become the most popular way of distinguishing semantic and response conflict in recent studies (e.g., Chen et al., 2011; Chen, Lei, Ding, Li, & Chen, 2013a; Chen, Tang & Chen, 2013b; Jiang et al., 2015; van Veen & Carter, 2005). First introduced by De Houwer (2003), this method maps two color responses to the same response button (see Fig. 1), which allows for a distinction between stimulus–stimulus (lexico-semantic) and stimulus–response (response) conflict.

By mapping two response options onto the same response key (e.g., both ‘blue’ and ‘yellow’ are assigned to the ‘z’ key), certain stimuli combinations (e.g., when *blue* is printed in yellow) are purported to not involve competition at the level of response selection; thus, any interference during same-response trials is thought to involve only semantic conflict. Any additional interference on different-response incongruent trials (e.g., when *red* is printed in yellow and where both ‘red’ and ‘yellow’ are assigned to different response keys) is taken as an index of response conflict. Performance on congruent trials (sometimes referred to as identity trials when used in the context of the two-to-one color-response mapping paradigm, here after 2:1 paradigm) is compared to performance on same-response incongruent trials to reveal interference that can be attributed to only semantic conflict, whereas a different-response incongruent vs same-response incongruent trial comparison is taken as an index of response conflict. Thus, the main advantage of using same-response incongruent trials as an index of semantic conflict is that this approach claims to be able to remove all of the influence of response competition (De Houwer, 2003). Notably, according to some models of Stroop task performance same-response incongruent trials should not produce interference because they do not involve response conflict (Cohen, Dunbar & McCelland, 1990; Roelofs, 2003).

Despite providing a seemingly convenient measure of semantic and response conflict, the studies that have employed the 2:1 paradigm share one major issue—that of an inappropriate baseline (see MacLeod, 1992). Same-response incongruent trials have consistently been compared to congruent trials to index semantic conflict. However, congruent trials also involve facilitation (both response and semantic facilitation—see below for more discussion of this) and thus, the difference between these two trial types could simply be facilitation and not semantic interference, a possibility De Houwer (2003) alluded to in his original paper (see also Schmidt et al., 2018). And whilst same-response trials plausibly involve semantic conflict, they are also likely to involve response facilitation because despite being semantically incongruent, the two dimensions of **this type of** Stroop stimulus provide evidence towards the same response. This means that both same-response and congruent trials involve response facilitation. Therefore the difference between same-response and congruent trials would actually be semantic conflict (experienced on same-response trials) + semantic facilitation (experienced on congruent trials), not just semantic conflict. This also has ramifications for the difference between different-response and same-response trials since the involvement of response facilitation on same-response trials means that the comparison of these two trials types would actually be response conflict plus response facilitation, not just response conflict.

Hasshim and Parris (2014) explored this possibility by comparing same-response incongruent trials to non-color-word neutral trials. They reasoned that this comparison could reveal faster RTs to same-response incongruent trials thereby providing evidence for response facilitation on same-response trials. In contrast, it could also reveal faster RTs to non-color-word neutral trials, thus, would have provided evidence for semantic interference (and would indicate that whatever response facilitation is present is hidden by an opposing and greater amount of semantic conflict). Hasshim and Parris reported no statistical difference between the RTs of the two trial types and reported Bayes Factors indicating evidence in favor of the null hypothesis of no difference. This would suggest that, when using reaction time as the index of performance, same-response incongruent trials cannot be employed as a measure of semantic conflict since they are not different from non-color-word neutral trials. In a later study, the same researchers investigated whether the two-to-one color-response mapping paradigm could still be used to reveal semantic conflict when using a more sensitive measure of performance than RT (Hasshim & Parris, 2015). They attempted to provide evidence for semantic conflict using an oculomotor Stroop task and an early, pre-response pupillometric measure of effort, which had previously been shown to provide a reliable alternative measure of the potential differences between conditions (Hodgson et al., 2009). However, in line with their previous findings, they reported Bayes Factors indicating evidence for no statistical difference between the same-response incongruent trials and non-color-word neutral trials. These findings, therefore, suggest that the difference between same-response incongruent trials and congruent trials indexes facilitation on congruent trials, and that the former trials are not therefore a reliable measure of semantic conflict when reaction times or pupillometry are used as the dependent variable. Notably, Hershman and Henik (2020) included neutral trials in their study of the 2:1 paradigm, but did not report statistics comparing same-response and neutral trials (although they did report differences between same-response and congruent trials where the latter had similar RTs to their neutral trials) It is clear from their Fig. 1, however, that pupil sizes for neutral and same-response trials do begin to diverge at around the time the button press response was made. This divergence gets much larger ~ 500 ms post-response indicating that a difference between the two trial types is detectable using pupillometry. Importantly, however, Hershman and Henik employed repeated letter string as their neutral condition, which does not involve task conflict (see the section on task conflict below for more details). This means that any differences between their neutral trial and the same-response trial could be entirely due to task and not semantic conflict.

However, despite Hasshim and Parris consistently reporting no difference between same-response and non-color-word neutral trials, in an unpublished study, Lakhzoum (2017) has reported a significant difference between non-color-word neutral trials and same-response trials. Lakhzoum’s study contained no special modifications to induce a difference between these two trial types, and had roughly similar trial and participant numbers and a similar experimental set-up to Hasshim and Parris. Yet Lakhzoum observed the effect that Hasshim and Parris have consistently failed to observe. The one clear difference between Lakhzoum (2017), Hasshim and Parris (2014, 2015), however, was that Lakhzoum used French participants and presented the stimuli in French where Hasshim and Parris conducted their studies in English. A question for further research then is whether and to what extent language, including issues such as orthographic depth of the written script of that language, might modify the utility of same-response trials as an index of semantic conflict.

Indeed, even though the 2:1 paradigm is prone to limitations, more research is needed to assess its utility for distinguishing response and semantic conflict. Notably, in both their studies Hasshim and Parris used colored patches as the response targets (at least initially, Hasshim & Parris, 2015, replaced the colored patches with white patches after practice trials) which could have reduced the magnitude of the Stroop effect (Sugg & McDonald, 1994). Same-response trials cannot, for obvious reasons, be used with the commonly used vocal response as a means to increase Stroop effects (see Response Modes and varieties of conflict section below), but future studies could use written word labels, a manipulation that has also been shown to increase Stroop effects (Sugg & McDonald, 1994), and thus might reveal a difference between same-response incongruent and non-color-word neutral conditions. At the very least future studies employing same-response incongruent trials should also employ a neutral non-color-word baseline (as opposed to color patches used by Shichel & Tzelgov, 2018) to properly index semantic conflict and should avoid the confounding issues associated with congruent trials (see also the section on Informational Facilitation below).

As noted above, same-response incongruent trials are also likely to involve response facilitation since both dimensions (word and color) provide evidence toward the same response. Since congruent trials and same-response incongruent trials both involve response facilitation, the difference between the two conditions likely represents semantic facilitation, not semantic conflict. As a consequence, indexing response conflict via the difference between different-response and same-response trials is also problematic. Until further work is done to clarify these issues, work applying the 2:1 color-response paradigm to understand the neural substrates of semantic and response conflicts (e.g., Van Veen & Carter, 2005) or wider issues such as anxiety (Berggren & Derakshan, 2014) remain difficult to interpret.

### Non-response set trials

Non-response set trials are trials on which the irrelevant color word used is not part of the response set (e.g., the word ‘orange’ in blue, where orange is not a possible response option and blue is; originally introduced by Klein, 1964). Since the non-response set color word will activate color-processing systems, interference on such trials has been interpreted as evidence for conflict occurring at the semantic level. These trials should in theory remove the influence of response conflict because the irrelevant color word is not a possible response option and thus, conflict at the response level is not present. The difference in performance between the non-response set trials and a non-color-word neutral baseline condition (e.g., the word ‘table’ in red) is taken as evidence of interference caused by the semantic processing of the irrelevant color word (i.e., semantic conflict). In contrast, response conflict can be isolated by comparing the difference between the performance on incongruent trials and the non-response set trials. This index of response conflict has been referred to as the response set effect (Hasshim & Parris, 2018; Lamers et al., 2010) or the response set membership effect (Sharma & McKenna, 1998) and describes the interference that is a result of the irrelevant word denoting a color that is also a possible response option. The aim of non-response set trials is to provide a condition where the irrelevant word is semantically incongruent with the relevant color such that the resultant semantic conflict is the only form of conflict present.

It has been argued that the interference measured using non-response set trials, the non-response set effect, is an indirect measure of response conflict (Cohen et al., 1990; Roelofs, 2003) and is, thus, not a measure of semantic conflict. That is, the non-response set effect results from the semantic link between the non-response set words and the response set colors and indirect activation of the other response set colors leads to response competition with the target color. As far as we are aware there is no study that has provided or attempted to provide evidence that is inconsistent with this argument. Thus, for non-response set trials to have utility in distinguishing response and semantic conflict, future research will need to evidence the independence of these types of conflict in RTs and other dependent measures.

### Semantic-associative trials

Another method that has been used to tease apart semantic and response conflict employs words that are semantically associated with colors (e.g., sky-blue, frog-green). In trials of this kind (e.g., *sky* printed in green), first introduced by Klein (1964), the irrelevant words are semantically related to each of the response colors. Recall that for Klein this was a way of investigating different magnitudes of response conflict (the indirect response conflict interpretation). Indeed, the notion of comparing RTs on color-associated incongruent trials to those on color-neutral trials to specifically isolate semantic conflict (i.e., so-called “sky-put” design) was first suggested by Neely and Kahan (2001). It was later actually empirically implemented by Manwell, Roberts and Besner (2004) and used since in multiple studies investigating Stroop interference (e.g., Augustinova & Ferrand, 2014; Risko et al., 2006; Sharma & McKenna, 1998; White et al., 2016).

Interference observed when using semantic associates tends to be smaller than when using non-response set trials (Klein, 1964; Sharma & McKenna, 1998). This suggests that semantic associates may not capture semantic interference in its entirety (or alternatively that non-response set trials involve some response conflict). Sharma and McKenna (1998) postulated that this is because non-response set trials involve an additional level of semantic processing which, following Neumann (1980) and La Heij, Van der Heijdan, and Schreuder (1985), they called semantic relevance (due to the fact that color words are also relevant in a task in which participants identify colors). It is, however, also the case that smaller interference observed with semantic associates compared to non-response set trials can be conceptualized simply as less semantic association with the response colors for non-color words (sky-blue) than for color words (red–blue).

As with non-response set trials, it is unclear whether semantic associates exclude the influence of response competition because they too can be modeled as indirect measures of response conflict (e.g., Roelofs, 2003). Since semantic-associative interference could be the result of the activation of the set of response colors to which they are associated (for instance when *sky* in red activates competing response set option blue), it does not allow for a clear distinction between semantic and response processes. In support of this possibility, Risko et al. (2006) reported that approximately half of the semantic-associative Stroop effect is due to response set membership and therefore response level conflict. The raw effect size of pure semantic-associative interference (after interference due to response set membership was removed) in their study was only between 6 ms (manual response, 112 participants) and 10 ms (vocal response, 30 participants).

When the same group investigated this issue with a different approach (i.e., ex-Gaussian analysis), their conclusions were quite different. White and colleagues (2016) found the semantic Stroop interference effect (difference between semantic-associative and color-neutral trials) in the mean of the normal distribution (mu) and in the standard deviation of the normal distribution (sigma), but not the tail of the RT distribution (tau). This finding was different from past studies that found standard Stroop interference in all three parameters (see, e.g., Heathcote et al., 1991). Therefore, White and colleagues reasoned that the source of the semantic (as opposed standard) Stroop effect is different such that the interference associated with response competition on standard color-incongruent trials (that is to be seen in tau) is absent in incongruent semantic associates. However, White et al. only investigated semantic conflict. A more recent study that considered both response and semantic conflict in the same experiment found they influence similar portions of the RT distribution (Hasshim, Downes, Bate, & Parris, 2019), suggesting that ex-Gaussian analysis cannot be used to distinguish the two types of conflict.

Interestingly, Schmidt and Cheesman (2005) explored whether semantic-associative trials involve response conflict by employing the 2:1 paradigm depicted above. With the standard Stroop stimuli, they reported the common differences between same- and different-response incongruent trials (that are thought to indicate response conflict) and between congruent and same-response incongruent (that are thought to indicate semantic conflict in the 2:1 paradigm). However, with semantic-associative stimuli they only observed an effect of semantic conflict a finding that differs from that of Risko et al. (2006) whose results indicate an effect of response conflict with semantic-associative stimuli. But, as already noted, the issues associated with employing just congruent trials as a baseline in the 2:1 paradigm and the potential response facilitation on same-response trials lessens the interpretability of this result.

Complicating matters further still, Lorentz et al. (2016) showed that the semantic-associative Stroop effect is not present in reaction time data when response contingency (a measure of how often an irrelevant word is paired with any particular color) is controlled by employing two separate contingency-matched non-color-word neutral conditions (but see Selimbegovic, Juneau, Ferrand, Spatola & Augustinova, 2019). There was, however, evidence for Stroop facilitation with these stimuli and for interference effects in the error data. Nevertheless, studies utilizing semantic-associative stimuli that have not controlled for response contingency might not have accurately indexed semantic-associative interference. Future research should focus on assessing the magnitude of the semantic-associative Stroop interference effect after the influences of response set membership and response contingency have been controlled.

Levin and Tzelgov (2016) also reported that they failed to observe the semantic-associative Stroop effect across multiple experiments using a vocal response (in both Hebrew and Russian). Only when the semantic associations were primed via a training protocol were semantic-associative Stroop effects observed, although they were not able to consistently report evidence for the null hypothesis of no difference. They subsequently argued that the semantic-associative Stroop effect is probably present but is a small and “unstable” contributor to Stroop interference. This is a somewhat surprising conclusion given the small but consistent effects reported by others with a vocal response (Klein, 1964; Risko et al., 2006; Scheibe et al., 1967; White et al., 2016; see Augustinova & Ferrand, 2014, for a review). However, it seems reasonable to conclude that the semantic-associative Stroop effect is not easily observed, especially with a manual response (e.g., Sharma & McKenna, 1998).

Finally, any observed semantic-associative interference could be interpreted as being an indirect measure of response competition (even after factors such as response set membership and response contingency are controlled). Indeed, the colors associated with the semantic-associative stimuli are also linked to the response set colors (Cohen et al., 1990; Roelofs, 2003) and thus, semantic associates do not generate an unambiguous measure of semantic conflict, at least when only RTs are used. Thus, it seems essential for future research to investigate this issue with additional, and perhaps more refined indicators of response processing such as EMGs.

### Semantics as distance on the electromagnetic spectrum

Klopfer (1996) demonstrated that RTs were slower when both dimensions of the Stroop stimulus were closely related on the electromagnetic spectrum. The electromagnetic spectrum is the range of frequencies of electromagnetic radiation and their wavelengths including those for visible light. The visible light portion of the spectrum goes from red with the shortest and violet with the longest wavelengths with Orange, Yellow, Green and Blue (amongst others) in between. The Stroop effect has been reported to be larger when the color and word dimensions of the Stroop stimulus are close on the spectrum (e.g., *blue* in green) compared to when the colors were distantly related (e.g., *blue* in red; see also Laeng et al., 2005, for an effect of color opponency on Stroop interference). In other words, Stroop interference is greater when the semantic distance between the color denoted by the word and the target color in “color space” is smaller, making it seemingly difficult to argue that semantic conflict does not contribute to Stroop interference. However, Kinoshita, Mills, and Norris (2018) recently failed to replicate this electromagnetic spectrum effect indicating that more research is needed to assess whether this is a robust effect. Even if replicated, however, this manipulation cannot escape the interpretation of semantic conflict as being the indirect indexing of response conflict. Therefore, these replications also call for additional indicators of response processing or the lack of thereof.

### Can we distinguish the contribution of response and semantic processing?

Perhaps due to the past competition between early and late selection, single-stage accounts of Stroop interference (Logan & Zbrodoff, 1998; MacLeod, 1991) response and semantic conflict have historically been the most studied and, therefore, compared types of conflict. For instance, there is a multitude of studies indicating that semantic conflict is often preserved when response conflict is reduced by experimental manipulations including hypnosis-like suggestion (Augustinova & Ferrand, 2012), priming (Augustinova & Ferrand, 2014), Response–Stimulus Interval (Augustinova et al., 2018a), viewing position (Ferrand & Augustinova, 2014a) and single letter coloring (Augustinova & Ferrand, 2007; Augustinova et al., 2010, 2015, 2018a, 2018b). This dissociative pattern (i.e., significant semantic conflict while response conflict is reduced or even eliminated) is often viewed as indicating two qualitatively distinct types of conflict, suggesting that these manipulations result in response conflict being prevented. However, these studies have commonly employed semantic-associative conflict which could be indirectly measuring response conflict and it could, therefore, be argued that it is not the type of conflict but simply residual response conflict that remains (Cohen et al., 1990; Roelofs, 2003). Therefore, it still remains plausible that the dissociative pattern simply indicates quantitative differences in response conflict.

As we have discussed in this section, interference generated by both non-response trials and trials that manipulation proximity on the electromagnetic spectrum are prone to the same limitations. The 2:1 paradigm is a paradigm that could in principle remove response conflict from the conflict equation, but the issues surrounding this manipulation need to be further researched before we can be confident of its utility. Therefore, at this point, it seems reasonable to conclude that published research conducted so far with additional color-incongruent trial types (same-response, non-response, or semantic-associative trials) does not permit the unambiguous conclusion that the informational conflict generated by standard color-incongruent trials (word ‘red’ presented in blue) can be decomposed into semantic and response conflicts. More than ever then, cumulative evidence from more time- and process-sensitive measures are required.

## Other types of informational conflict: considering the role of phonological processing and word frequency

Whilst participants are asked to ignore the irrelevant word in the color-word Stroop task, it is clear that their attempts to do so are not successful. If word processing proceeds in an obligatory fashion such that before accessing the semantic representation of the irrelevant word, the letters, orthography, and phonology are also processed, interference could happen at these levels of processing. But, as anticipated by Klein (1964), just because the word is processed at these levels does not mean that each leads to level-specific conflict. To determine whether or not these different levels of processing also independently contribute to Stroop interference, various trial types and manipulations have been employed that have attempted to dissociate pre-semantic levels of processing. The most notable methods are: (1) phonological overlap between the irrelevant word and color name; (2) the use of pseudowords; and (3) manipulation of word frequency. This section attempts to identify whether pre-semantic processing of the irrelevant word reliably leads to conflict (or facilitation) at levels other than response output.

### Phonological overlap between word and color name

A study by Dalrymple-Alford (1972) presented evidence for solely phonological interference in the Stroop task. Dalrymple-Alford manipulated the phonemic overlap between the irrelevant word and color name. For example, if the color to be named was red, the to-be-ignored word would be *rat* (sharing initial phoneme) or *pod* (sharing the end phoneme) or a word that shares no phoneme at all (e.g., *fit*). Dalrymple-Alford reported evidence for greater interference at the initial letter than at the end letter position (similar effects were observed for facilitation). Using a more carefully designed set of stimuli (originally created by Coltheart et al., 1999, who focused on just facilitation), Marmurek et al. (2006) also showed greater interference and facilitation at the initial letter position than the end letter position; although, in their study effects at the end letter position did not reach significance. This paradigm represents a direct measure of phonological processing that, importantly, does not have a semantic component (other than the weak conflict that would result from the activation of two semantic representations with unrelated meanings). However, in line with the interpretation by Coltheart et al. (1999), Marmurek and colleagues argued it was evidence for phonological processing of the irrelevant word that either facilitates or interferes with the production of the color name at the response output stage (see also Parris et al., 2019a, 2019b, 2019c; Regan, 1978; Singer et al., 1975). Thus, whilst the word is processed phonologically, the only phonological representation with which the resulting representation could compete is that created during the phonological encoding of the color name, which would only be produced at later response processing levels. In sum, it is not possible to conclude in favor of qualitatively different conflict (or facilitation) other than that at the response level using this approach.

### Pseudowords

A pseudoword is a non-word that is pronounceable (e.g., *veglid*). In fact, some real words are so rare (e.g., *helot*, *eft*) that to most they are equivalent to pseudowords. As noted above, Klein (1964) used rare words in the Stroop task and showed that they interfered less than higher-frequency words but more than consonant strings (e.g., *GTBND*). Both Burt’s (2002) and Monsell et al.’s (2001) studies later supported the finding that pseudowords result in more interference than consonant strings. In recent work, Kinoshita et al. (2017) asked what aspects of the reading process is triggered by the irrelevant word stimulus to produce interference in the color-word Stroop task. They compared performance on five types of color-neutral letter strings to incongruent words. They included real words (e.g., *hat*), pronounceable non-words (or pseudowords; e.g., *hix*), consonant strings (e.g., *hdk*), non-alphabetic symbol strings (e.g., *&@£*), and a row of Xs. They reported that there was a word-likeness or pronounceability gradient with real words and pseudowords showing an equal amount of interference (with interference increasing with string length) and more than that produced by the consonant strings. Consonant strings produced more interference than the symbol strings and the row of Xs which did not differ from each other. The absence of the lexicality effect (defined by color-neutral real words producing more interference than pseudowords) was explained by Kinoshita and colleagues as being a consequence of the pre-lexically generated phonology from the pronounceable irrelevant words interfering with the speech production processes involved in naming the color. Under this account, the process of phonological encoding (the segment-to-frame association processes in articulation planning) of the color name must be slowed by the computation of phonology that occurs independent of lexical status (because it happens with pronounceable pseudowords). Notably, the authors reported evidence for pre-lexically generated phonology when participants responded vocally (by saying aloud the color name), but not when participants responded manually (by pressing a key that corresponds to the target color) suggesting the effects were the result of the need to articulate the color name.

Some pseudowords can sound like color words (e.g., bloo), and are known as pseudohomophones. Besner and Stolz (1998) employed pseudohomophones as the irrelevant dimension, and found substantial Stroop effects when compared to a neutral baseline (see also Lorentz et al., 2016; Monahan, 2001) suggesting that there is phonological conflict in the Stroop task. However, pseudohomophones do not involve only phonological conflict since they contain substantial orthographic overlap with their base words (e.g., *bloo*, *yeloe*, *grene*, *wred*) and will likely activate the semantic representations of the colors indicated by the word via their shared phonology. In short, interference produced by pseudohomophones could result from phonological, orthographic, or semantic processing but also and importantly it can still simply result from response conflict (see also Tzelgov et al., 1996, work on cross-script homophones which shows phonologically mediated semantic/response conflict, but not phonological conflict).

Taken together, this work shows a clear effect of phonological processing of the irrelevant word on Stroop task performance; and one that likely results from the pre-lexical phonological processing of the irrelevant word. Again, however, it is unclear whether the resulting competition arises at the pre-lexical level (suggesting the color name’s pre-lexical phonological representation is unnecessarily activated) or whether phonological processing of the irrelevant word leads to phonological encoding of that word that then interferes with the phonological encoding of the relevant color name. The latter seems more likely than the former.

### High- vs. low-frequency words

In support of the notion that non-semantic lexical factors contribute to Stroop effects, studies have shown an effect of the word frequency of non-color-related words on Stroop interference. Word frequency refers to the likelihood of encountering that word in reading and conversation. It is a factor that has long been known to contribute to word reading latency, and given that color words tend to be high-frequency words, it is possible word frequency contributes to Stroop effects. Whilst the locus of word frequency effects in word reading are unclear, it is known that it takes longer to access lexico-semantic (phonological/semantic) representations of low-frequency words (Gherhand & Barry, 1998, 1999; Monsell et al., 1989).

According to influential models of the Stroop task, the magnitude of Stroop interference is determined by the strength of the connection between the irrelevant word and the response output level (Cohen et al., 1990; Kalanthroff et al., 2018; Zhang et al., 1999). Since high-frequency words are by definition encountered more often, their strength of connection to the response output level would be higher than that for low-frequency words. This leads to the prediction that color-naming times should be longer when the distractor word is of a higher frequency. Evidence in support of this has been reported by Klein (1964), Fox et al. (1971) and Scheibe et al. (1967). However, Monsell et al. (2001) pointed out methodological issues in these older studies that could have confounded the results. First, these previous studies employed the card presentation version of the Stroop task in which the items from each stimulus condition (e.g., all the high-frequency words) are placed on different cards and the time taken to respond to all the items on one card is recorded. This method, it was argued, could result in the adoption of different response criteria for the different cards and permits previews of the next stimulus which could result in overlap of processing. Second, Monsell et al. noted that these studies employed a limited set of 4–5 stimuli in each condition which were repeated numerous times on each card, potentially leading to practice effects that would potentially nullify any effects of word frequency. After addressing these issues, Monsell et al. (2001) reported no effects of word frequency on color-naming times, although there was a non-significant tendency for low-frequency words to result in more interference than high-frequency words. With the same methodological control as Monsell et al., but with a greater difference in frequency between the high and low conditions, Burt (1994, 1999, 2002) has repeatedly reported that low-frequency words produce significantly more interference than high-frequency words (findings recently replicated by Navarrete et al., 2015). A recent study by Levin and Tzelgov (2016) also reported more interference to low-frequency words although their effects were not consistent across experiments, a finding that could be attributed to their use of a small set of words for each class of words.

The repeated finding of greater interference for low-frequency words is consistent with the notion that word frequency contributes to determining response times in the Stroop task, but is inconsistent with predictions from models of the class exemplified by Cohen et al. (1990). The finding of larger Stroop effects for lower-frequency words provides a potent challenge to the many models based on the Parallel Distributed Processing (PDP) connectionist framework (Cohen et al., 1990; Kalanthroff et al., 2018; Kornblum et al., 1990; Kornblum & Lee, 1995; Zhang & Kornblum, 1998; Zhang et al., 1999; see Monsell et al., 2001 for a full explanation of this). As noted, these models would argue, on the basis of a fundamental tenet of their architectures, that higher-frequency words should produce greater interference because they have stronger connection strengths with their word forms. Notably, whilst unsupported by later studies, the lack of an effect of word frequency in Monsell et al.’s data led them to the conclusion that there was another type of conflict involved in the Stroop task, called task conflict. It is to the topic of task conflict that we now turn.

## Task conflict

The presence of task conflict in the Stroop task was first proposed in MacLeod and MacDonald’s (2000) review of brain imaging studies (see also Monsell et al., 2001; see Littman et al., 2019, for a mini review). The authors proposed its existence because the anterior cingulate cortex (ACC) appeared to be more activated by incongruent and congruent stimuli when compared to repeated letter neutral stimuli such as xxxx (e.g., Bench et al., 1993). MacLeod and MacDonald suggested that increased ACC activation by congruent and incongruent stimuli reflects the signaling the need for control recruitment in response to task conflict. Since task conflict is produced by the activation of the mental machinery used to read, interference at this level occurs with any stimulus that is found in the mental lexicon. Studies have used this logic to isolate task conflict from informational conflict (e.g., Entel & Tzelgov, 2018).

### Congruent trials, proportion of repeated letter strings trials and negative facilitation

In contrast to color-incongruent trials that are thought to produce both task and informational conflicts, color-congruent trials are only thought to produce task conflict. Conflict of any type, by definition, increases response times and thus, congruent trial reaction times can be expected to be longer than those on trials that do *not* activate a task set for word reading. Repeated color patches, symbols or letters (e.g., ■■■, xxxx or ####) have, therefore, been introduced as a baseline for such a comparison. Indeed, these trials are not expected to generate task conflict as they do not activate an item in the mental lexicon. The difference between these non-linguistic baselines and congruent trials would therefore represent a measure of task conflict, and has been referred to as negative facilitation. However, a common finding in such experiments is that congruent trials still produce faster RTs than neutral non-word stimuli or positive facilitation (Entel et al., 2015; see also Augustinova et al., 2019; Levin & Tzelgov, 2016, Shichel & Tzelgov, 2018), indicating that task conflict is not fully measured under such conditions. Goldfarb and Henik (2007) reasoned that this is likely due to the fact that faster responses on congruent trials compared to a non-linguistic baseline results when task conflict control is highly efficient, permitting the expression of positive facilitation.

To circumvent this issue, they attempted to reduce task conflict control by increasing the proportion of non-word neutral trials (repeated letter strings) to 75% (see also Kalanthroff et al., 2013). Increasing the proportion of non-word neutral trials would create the expectation for a low task conflict context and so task conflict monitoring would effectively be offline. In addition to increasing the proportion of non-word neutral trials, on half of the trials, the participants received cues that indicated whether the following stimulus would be a non-word or a color word, giving another indication as to whether the mechanisms that control task conflict should be activated. For non-cued trials, when presumably task conflict control was at its nadir, and therefore task conflict at its peak, RTs were slower for congruent trials than for non-word neutral trials, producing a negative facilitation effect. Goldfarb and Henik (2007) suggested that previous studies had not detected a negative facilitation effect because resolving task conflict for congruent stimuli does not take long, and thus, as mentioned above, the effects of positive facilitation had hidden those of negative facilitation. In sum, by reducing task control both globally (by increasing the proportion of neutral trials) and locally (by adding cues to half of the trials), Goldfarb and Henik were able to increase task conflict enough to demonstrate a negative facilitation effect; an effect that has been shown to be a robust and prime signature of task conflict (Goldfarb & Henik, 2006, 2007; Kalantroff et al., 2013).

Steinhauser and Hübner (2009) manipulated task conflict control by combining the Stroop task with a task-switching paradigm. In this paradigm participants switch between color naming and reading the irrelevant word (see Kalanthroff et al., 2013, for a discussion on task switching and task conflict). Thus, the two task sets are active in this task context. This means that during color-naming Stroop trials, the word dimension of the stimulus will be more strongly associated with word processing than it otherwise would. This would have the effect of increasing the conflict between the task set for color naming and the task set of word reading. Steinhauser and Hübner (2009) found that under these experimental conditions, participants performed worse on congruent (and incongruent) trials than they did on the non-word neutral trials, evidencing negative facilitation, the key marker of task conflict. These results showing increasing task conflict when there is less control over the task set for word reading on color-naming trials reaffirmed Goldfarb and Henik’s (2007) findings that showed that reducing task control on color-naming trials leads to task conflict.

Whilst both of the above methods are useful in showing that task conflict can influence the magnitude of Stroop interference and facilitation, both manipulations result in magnifying task conflict (and likely other forms of conflict) to levels greater than is present when such targeted manipulations are not used.

### Repeated letter strings without a task conflict control manipulation

As has been noted, task conflict appears to be present whenever the irrelevant stimulus has an entry in the lexical system. Consequently, studies have used the contrast in mean color-naming latencies between color-neutral words and repeated letter strings to index task conflict (Augustinova et al., 2018a; Levin & Tzelgov, 2016). However, Augustinova et al. argued that both of these stimuli might include task conflict in different quantities. This is because the processing activated by a string of repeated letters (e.g., xxx) stops at the orthographic pre-lexical level, whereas the one activated by color-neutral words (e.g., dog) proceeds through to access to meaning (see also Augustinova et al., 2019; Ferrand et al., 2020), and as such the latter might more strongly activate the task set for word reading. Augustinova et al. (2019) reported task conflict (color-neutral—repeated letter strings) with vocal responses but not manual responses. Likewise, in a manual response study, Hershman et al. (2020) reported that repeated letter strings did not differ in terms of Stroop interference relative to symbol strings, consonant strings and color-neutral words. All were responded to more slowly than congruent trials, however, evidencing facilitation on congruent trials. Levin and Tzelgov (2016) compared vocal response color-naming times of repeated letter strings and shapes and found that repeated letter strings had longer color-naming times indicating some level of extra conflict with repeated letter strings, which they referred to as orthographic conflict, but which could also be expected to activate a task set for word reading. The implication of this work is that whilst repeated letter strings can be used as a baseline against which to measure task conflict relative to color-neutral words, they are likely to be useful mainly with vocal responses (Augustinova et al., 2019), and moreover can be expected to lead to some level of task conflict (Levin & Tzelgov, 2016).

For a purer measure of task conflict, when eschewing manipulations needed to produce negative facilitation, future research would do better to compare response times for color-neutral stimuli with those for shapes whilst employing a vocal response (Levin & Tzelgov, 2016; see Parris et al., 2019a, 2019b, 2019c, who reported no difference between color-neutral stimuli and unnamable/novel shapes with a manual response in an fMRI experiment). This does not mean, however, that task conflict is not measureable with manual responses in designs that eschew manipulations that produce negative facilitation: Continuing with their exploration of Stroop effects in pupillometric data Hershman et al. (2020) reported that pupil size data revealed larger pupils to congruent than to repeated letter strings (and also symbol strings, consonant strings and non-color-related words); in other words, they reported negative facilitation.

### Does task conflict precede informational conflict?

The studies discussed above also suggest that task conflict occurs earlier than informational conflict. Hershman and Henik (2019) recently provided evidence that supports this supposition. Using incongruent, congruent and a repeated letter string baseline, but without manipulating the task conflict context in a way that would produce negative facilitation, Hershman and Henik observed a large interference effect and small non-significant, positive facilitation. However, the authors also recorded pupil dilations during task performance and reported both interference and negative facilitation (pupils were smaller for the repeated letter string condition than for congruent stimuli). Importantly, the pupil data began to distinguish between the repeated letter string condition and the two word conditions (incongruent and congruent) up to 500 ms before there was divergence between the incongruent and congruent trials. In other words, task conflict appeared earlier than informational conflict in the pupil data.

If it is not firmly established that task conflict comes before informational conflict on a single trial, recent research has shown that it certainly seems to come first developmentally. By comparing performance in 1st, 3rd and 5th graders, Ferrand and colleagues (2020) showed that 1st graders experience smaller Stroop interference effects (even when controlling for processing speed differences) compared to 3rd and 5th graders. Importantly, whereas the Stroop interference effect in these older children is largely driven by the presence of response, semantic and task conflict, in the 1st graders (i.e., pre-readers) this interference effect was entirely due to task conflict. Indeed, these children produced slower color-naming latencies for all items using words as distractors compared to repeated letter strings, without being sensitive to color-(in)congruency and to the informational (phonological, semantic or. response) conflict that it generates. The finding of task conflict’s developmental precedence is consistent with the idea that visual expertise for letters (as evidence by aforementioned N170 tuning for print) is known to be present even in pre‐readers (Maurer et al., 2005).

### A model of task conflict

Kalanthroff et al. (2018) presented a model of Stroop task performance that is based on processing principles of Cohen and colleagues’ models (Botvinick et al., 2001; Cohen et al, 1990). What is unique about their model is the role proactive (intentional, sustained) control plays in modifying task conflict (see Braver, 2012). When proactive control is strong, bottom-up activation of word reading is weak, and top-down control resolves any remaining task competition rapidly. Conversely, when proactive control is weak, bottom-up information can activate task representations more readily leading to greater task conflict. According to their model, the presence of task conflict inhibits all response representations, effectively raising the response threshold and slowing responses. This raising of the response threshold would not happen for repeated letter string trials (e.g., xxxx) because the task unit for word reading would not be activated. Since responses for congruent trials would be slowed, negative facilitation results. To control task conflict when it arises, Kalanthroff et al. (2018) argued that due to the low level of proactive control, reactive control is triggered to resolve task conflict via the weak top-down input from the controlling module in the Anterior Cingulate Cortex. Thus, in contrast to Botvinick et al.’s (2001) model, reactive control is triggered by weak proactive control, not the detection of informational conflict. When proactive control is high, there is no task conflict, and the reactive control mechanism is not triggered, and the response convergence at the response level leads to response facilitation which can be fully expressed. Since task conflict control is not reliant on the presence of intra-trial informational conflict, and it is not resolved at the response output level, it is resolved by an independent control mechanism. Thus, the Kalanthroff et al. model predicts the independent resolution of response and task conflict.

In sum, task conflict has been shown to be an important contributor to both Stroop interference and Stroop facilitation effects. Task conflict can result in the reduction of the Stroop facilitation effect, increased Stroop interference, and in its more extreme form, it can produce negative facilitation (RTs to congruent trials are longer than those to a non-word neutral baseline). A concomitant decrease in Stroop facilitation and increase in Stroop interference (or vice versa) is also another potential marker of task conflict (Parris, 2014), although since a reduced Stroop facilitation and an increased Stroop interference can be produced by other mechanisms (i.e., decreased word reading/increased attention to the color dimension and increased response conflict, respectively), at this point, negative facilitation is clearly the best marker of task conflict (in RT or pupil data; Hershman & Henik, 2019). Kalanthroff et al. (2018) have argued that task conflict is a result of low levels of proactive control. However, more work is perhaps needed to identify what triggers activation of the task set for word reading and how types of informational conflict might interact with task conflict. Levin and Tzelgov (2016) describe informational conflict as being an “episodic amplification of task interference” (p3), where task conflict is a marker of the automaticity of reading and informational conflict the effect of dimensional overlap between stimuli and responses. With recent evident suggesting readability is a key factor in producing task conflict (Hershman et al., 2020), task conflict is possibly closely related to the ease with which a string of letters is phonologically encoded, its pronounceability (Kinoshita et al., 2017), suggesting a link between task and phonological conflict. Indeed, Levin and Tzelgov (2016) associated the orthographic and lexical components of word reading with task conflict. However, it is unclear how phonological processing is categorized in their framework and importantly how facilitation effects are accounted for under such a taxonomy.

## Informational facilitation

As already mentioned, Dalrymple-Alford and Budayr (1966, Exp. 2) were the first to report a facilitation effect of the irrelevant word on color naming (see also Dalrymple-Alford, 1972 for coining the term). Since then, the Stroop facilitation effect has become an oft-present effect in Stroop task performance and is usually measured by the difference in color-naming performance on non-color-word trials and color-congruent trials. However, the use of congruent trials is, more than any other trial type, fraught with confounding issues. As amply developed in the previous section, when task conflict is high, congruent word trial RTs can actually be longer than non-color-word trial RTs eliminating the expression of positive facilitation in the RT data and even producing negative facilitation (Goldfarb & Henik, 2007). Indeed, perhaps the first record of task conflict in the Stroop literature, Heathcote et al. (1991) reported that whilst the arithmetic mean difference between color-congruent and color-neutral trial types reveals facilitation in the Gaussian portion of the RT distribution, it actually reveals interference in the tail of the RT distribution. In sum, congruent trial RTs are clearly influenced by processes that pull RTs in different directions. Moreover, it has been argued that Stroop facilitation effects are not true facilitation effects at all, in the sense that the faster RTs on congruent trials do not represent the benefit of converging information from the two dimensions of the Stroop stimulus (see below for a further discussion of this issue). Thus, before considering what levels of processing contribute to facilitation effects, we must first consider the nature of such effects.

### Accounting for positive facilitation

Since clear empirical demonstrations of task conflict being triggered by color-congruent trials were reported (see above), it has become difficult to consider the Stroop facilitation effect as a flip side of the Stroop interference (Dalrymple-Alford & Budayr, 1966). Stroop facilitation is often observed to be smaller, and less consistent, than Stroop interference (MacLeod, 1991) and this asymmetricity is largely dependent on the baseline used (Brown, 2011). Yet, this asymmetrical effect has been accounted for by models of the Stroop task via informational facilitation (i.e., without considering the opposing effect of task conflict). For example, in Cohen et al.’s (1990) model smaller positive facilitation is accounted for via a non-linear activation function which imposes a ceiling effect on the activation of the correct response—in other words, double the input (convergence) does not translate into double the output (Cohen et al., 1990).

MacLeod and McDonald (2000) and Kane and Engle (2003) have argued that the facilitating effect of the color-congruent irrelevant word is not true facilitation from any level of processing and is instead the result of ‘inadvertent reading’. That is, on some color-congruent trials, participants use only the word dimension to generate a response, meaning that these responses would be 100 ms–200 ms faster than if they were color naming (because word reading is that much faster than color naming). The argument is that it happens on only the occasional congruent trial (because of the penalty (error or large RTs) that would result from carrying it over to incongruent trials). Doing this occasionally would equate to the roughly 25 ms Stroop facilitation effect observed in most studies and would explain why facilitation is generally smaller than interference. Since the color-naming goal is not predicted to be active on these occasional congruent trials, it implies that only the task set for word reading is active, and hence the absence (or a large reduction) of task conflict, which fits with the finding of more informational facilitation in low task conflict contexts. Inadvertent reading would also be expected to produce facilitation in the early portion of the reaction time distribution (as supported by Heathcote et al.’s findings).

Roelofs (2010) argued, however, that with cross-language stimuli presented to bilingual participants, words cannot be read aloud to produce facilitation between languages (i.e., the Dutch word *Rood*—meaning ‘red’—cannot be read aloud to produce the response ‘red’ by Dutch–English bilinguals). Roelofs (2010) asked Dutch–English bilingual participants to name color patches either in Dutch or English whilst trying to ignore contiguously presented Dutch or English words. Given that informational facilitation effects were observed both within and between languages, Roelofs argued that the Stroop facilitation effect cannot be based on inadvertent reading. However, whilst Rood (Red), Groen (Green), and Blau (Blue) are not necessarily phonologically similar to their English counterparts, they clearly share orthographic similarities, which could produce facilitation effects (including semantic facilitation). Still, Roelofs observed large magnitudes of facilitation effects rendering it less likely that facilitation was based solely on orthography, although this was primarily when the word preceded the onset of the color patch. There were indeed relatively small facilitation effects when the word and color were presented at the same time. Nevertheless, the inadvertent reading account also cannot easily explain facilitation on semantic-associative congruent trials (see below for evidence of this) since the word does not match the response.

Another influence that can account for the facilitating effect of congruent trials is response contingency. Response contingency refers to the association between an irrelevant word and a response. In a typical Stroop task set-up, the numbers of congruent and incongruent trials are matched (e.g., 48 congruent/48 incongruent). Since in each congruent trial, there is only one possible word to pair with each color, it means that each color word is more frequently paired with its corresponding color (when the word red is displayed, there is a higher probability of its color being red). This would mean that responses on congruent trials would be further facilitated through learned word–response associations, and those on incongruent trials further slowed, by something other than and additional to the consequence of word processing (Melara & Algom, 2003; Schmidt & Besner, 2008). Indeed, it is as yet unclear as to whether informational facilitation would remain if facilitative effects of response contingency were controlled. Therefore, future studies are needed to address this still open issue (see Lorentz et al., 2016 for this type of endeavor but with semantic associates).

### Decomposing informational facilitation

Perhaps because it has been perceived as the lesser, and less stable effect, the Stroop facilitation effect has not been explored as much as the Stroop interference effect in terms of potential varieties of which it may be comprised (Brown, 2011). Coltheart et al. (1999) have shown that when the irrelevant word and the color share phonemes (e.g., *rack* in red, *boss* in blue), participants are faster to name the color than when they do not (e.g., *hip* in red, *mock* in blue). Given that none of the words used in their experiment contained color relations, their effect was likely entirely based on phonological facilitation (see also Dennis & Newstead, 1981; Marmurek et al., 2006; Parris et al., 2019a, 2019b, 2019c; Regan, 1979). Notably, effects such as this could not be explained by either the inadvertent reading nor response convergence accounts of Stroop facilitation and could not have resulted from response contingency (whilst any word in red, green or blue would have a greater chance of beginning with an ‘r’, ‘g’ and ‘b’ than any other letter respectively, there were three times as many trials in which the words did not begin with those letters). It is possible, however, that phonological facilitation operates on a different mechanism to semantic and response facilitation effects.

To the best of our knowledge only four published studies have explored this variety of informational facilitation directly. Dalrymple-Alford (1972) reported a 42 ms semantic-associative facilitation effect (non-color-word neutral—semantic-associative congruent) and a 67 ms standard facilitation effect (non-color-word neutral—congruent) suggesting a response facilitation effect of 25 ms (see Glaser & Glaser, 1989; and Mahon et al., 2012, for replications of this effect). Interestingly, however, when compared to a letter string baseline (e.g., xxxx), the congruent semantic associates actually produced interference—a finding implicating an influence of task conflict. More recently, Augustinova et al. (2019) reported semantic (11 ms) and response (39 ms) facilitation effects with vocal responses but only semantic facilitation (14 ms) with manual responses (response facilitation was a non-significant 7 ms). Interestingly, the comparison between the letter string baseline and congruent semantic associates produced 9 ms facilitation with the manual response, but 33 ms interference with the vocal response suggesting a complex relationship between response mode, semantic facilitation and task conflict. Indeed, exactly like color-congruent items discussed above, both congruent semantic-associative trials and their color-neutral counterpart with no facilitatory components still involve task conflict.

These (potentially) isolable forms of facilitation are interesting, require further study, and have the potential to shed light on impairments in selective attention and cognitive control. Of particular interest is how these forms of facilitation are modified by the presence of various levels of task conflict. Nevertheless, as with semantic conflict, it is possible that apparent semantic facilitation effects result from links between the irrelevant dimension and the response set colors (Roelofs, 2003) meaning that they are response- and not semantically based effects. Therefore, other approaches are needed to tackle the issue of semantic (vs. response) facilitation. It might be useful to recall at this point that both Roelofs’ (2010) cross-language findings and the differences in reaction times between congruent and same-response trials (e.g., De Houwer, 2003) possibly result from semantic facilitation and so would not be helpful in this regard.

## Other evidence relevant to the issue of locus vs. loci of the Stroop effect

### Response modes and the loci of the Stroop effect

Responding manually (via keypress) in the Stroop task consistently leads to smaller Stroop effects when compared to responding vocally (saying the name aloud, e.g., Augustinova et al., 2019; McClain, 1983; Redding & Gerjets, 1977; Repovš, 2004; Sharma & McKenna, 1998). It has been argued that this is because each response type has differential access to the lexicon where interference is proposed to occur (Glaser & Glaser, 1989; Kinoshita et al., 2017; Sharma & McKenna, 1998). Indeed, smaller Stroop effects with manual (as opposed to vocal) responses has been attributed to one of its components (i.e., semantic conflict) being significantly reduced (Brown & Besner, 2001; Sharma & McKenna, 1998). Therefore, the manipulation of response mode has been used to address the issue of the locus of the Stroop effect.

In response to reports of failing to observe Stroop effects with manual responses (e.g., McClain, 1983), Glaser and Glaser (1989) proposed in their model that manual responses with color patches on the response keys could not produce interference because perception of the color and the response to it were handled by the semantic system with little or no involvement of the lexical system where interference was proposed to occur. However, based on the earlier translation models (e.g., Virzi & Egeth, 1985), Sugg and McDonald (1994) showed that Stroop interference was obtained with manual responses when the response buttons were labeled with written color words instead of colored patches. Sugg and McDonald argued that written label responses must have direct access to the lexical system.

Using written label manual responses, Sharma and McKenna (1998) tested Glaser and Glaser’s model and showed that response mode matters when considering the types of conflict that participants experience in the Stroop task. They reported that in contrast to vocal responses, manual responses produced no lexico-semantic interference as measured by comparing semantic-associative and non-color-word neutral trials, and by comparing non-response set trials with semantic-associative trials, although they did report a response set effect (response set—non-response set) with both vocal (spoken) and manual responses. Sharma and McKenna interpreted their results as being partially consistent with Glaser and Glaser’s model, suggesting that the types of conflict experienced in the Stroop task are different between response modes. However, Brown and Besner (2001) later re-analyzed the data from Sharma and McKenna and showed that if you do not only analyze adjacent conditions (with condition order determined by a priori beliefs about the magnitude of Stroop effects) and compare instead non-adjacent conditions such as non-response set and non-color-word neutral trials (the non-response set effect), semantic conflict is observed with a manual response.

Roelofs (2003) has theorized that interference with manual responses only occurs because verbal labels are attached to the response keys; such a position predicts that manual and vocal responses should lead to similar conflict and facilitation effects, but smaller overall effects with manual responses due to the proposed mediated nature of manual Stroop effects. Consistently, many studies have since reported robust interference effects including semantic conflict effects with manual responses using colored patch labels (as measured by non-response set—non-color-word neutral, e.g., Hasshim & Parris, 2018; or as measured by semantic-associative Stroop trials, e.g., Augustinova et al., 2018a). Parris et al., (2019a, 2019b), Zahedi, Rahman, Stürmer, & Sommer (2019) and Kinoshita et al. (2017) have reported data indicating that the difference between manual and vocal responses occurs later in the phonological encoding or articulation planning stage where vocal responses encourage greater phonological encoding than does the manual response (see Van Voorhis & Dark, 1995 for a similar argument).

Augustinova et al. (2019) have reported that the difference between manual and vocal responses is largely due to a larger contribution of response conflict with vocal responses. Yet, in addition they also reported a much larger contribution of task conflict with vocal responses. Notably, the contribution of both semantic conflict and semantic facilitation remained roughly the same for the response modes, whereas response facilitation increased dramatically (from non-significant 7 ms to 39 ms) with vocal responses indicating that response and semantic forms of facilitation are independent. Therefore, the research to date suggests that there are larger response- and task-based effects with vocal responses. Since negative facilitation was not used as a measure of performance in this study, which has been reported with manual responses (e.g., Goldfarb & Henik, 2007), one needs to be careful what conclusions are drawn about task conflict; nevertheless, task conflict does seem to contribute less to Stroop effects with manual responses under common Stroop task conditions in which task conflict control is not manipulated. Importantly, this only applies to response times. As already noted, Hershman and Henik (2019) reported no task conflict with manual responses but also showed that in the same participants pupil sizes changes revealed task conflict in the form of negative facilitation on the very same trials.

It is important that more research investigating how the make-up of Stroop interference might change with response mode is conducted, especially since other response modes such as typing (Logan & Zbrodoff, 1998), oculomotor (Hasshim & Parris, 2015; Hodgson et al., 2009) and mouse (Bundt, Ruitenberg, Abrahamse, & Notebaert, 2018) responses have been utilized. This is especially important given that a lesion to the ACC has been reported to affect manual but not vocal response Stroop effects (Turken & Swick, 1999). Up until very recently very little consideration has been given to how response mode might affect Stroop facilitation effects (Augustinova et al., 2019) so more research is needed to better understand the influence of response mode on facilitation effects. Indeed, as noted above models have proposed either the same or different processes underlying manual and vocal Stroop effects providing predictions that need to be more fully tested. Aside from issues surrounding measurement of the varieties of conflict and facilitation that underlie Stroop effects with manual and vocal responses, mitigating the conclusions that can be drawn from the work summarized in this section, it is interesting that the way we act on the Stroop stimulus can potentially change how it is processed.

### Beyond response selection: Stroop effects on response execution

So far, we have concentrated on Stroop effects that occur before response selection. However, it is also possible that Stroop effects could be observed after (or during) response selection. When addressing questions about the locus of the Stroop effect, some studies have questioned the commonly held assumption that there is modularity between response selection and response execution; that is, they have considered whether interference experienced at the level of response selection spills over into the actual motoric action of the effectors (e.g., the time it takes to articulate the color name) or whether interference is entirely resolved before then. Researchers have considered this possibility with vocal (measuring the time between the production of the first phoneme and the end of the last; Kello et al., 2000), type-written (measuring the time between the pressing of the first letter key and the pressing of the last letter key; Logan & Zbrodoff, 1998), oculomotor (measuring the amplitude (size) of the saccade (eye movement) to the target color patch; Hodgson, Parris, Jarvis & Gregory, 2009), and mouse movement (Bundt et al., 2018; Yamamoto, Incera & McLennan, 2016) responses.

In Hodgson et al.’s (2009) study, participants responded by making an eye movement to one of four color patches located in a plus-sign configuration around the centrally presented Stroop stimulus to indicate the font color of the Stroop stimulus. In two experiments, one in which the target’s color remained in the same location throughout the experiment and one in which the colors occupied a different patch location (still in the plus-sign configuration) on every trial, Stroop interference effects were observed on saccadic latency, but not on saccade amplitude or velocity indicating that all interference is resolved before a motor movement is made and, therefore, that Stroop interference does not affect response execution. Similar null effects on response execution were reported for type-written responses across four experiments by Logan and Zbrodoff (1998).

Kello et al. (2000) initially also observed no Stroop effects on vocal naming durations (the time it takes to actually vocalize the response). In a follow-up experiment, however, in which they introduced a response deadline of 575 ms, they observed Stroop congruency effects on response durations. This likely holds for the other studies on response execution mentioned here. Indeed, Hodgson et al. pointed out that they could not exclude the possibility that under some circumstances the spatial characteristics of saccades would also show effects on incongruent trials given previous work showing that increasing spatial separation between target and distractor stimuli leads to an increase in the effect of the distractor on characteristics of the saccadic response (Findlay, 1982; McSorley et al., 2004; Walker et al., 1997).

Bundt et al. (2018) recently reported a Stroop congruency effect on response execution times in a study requiring participants to use a computer mouse to point to the target patch on the screen. Response targets where all in the upper half of the computer screen and participants guided the mouse from a start position in the lower half of the screen. They observed this effect despite not separating the target and distractor or enforcing a response time deadline. The configuration differences, the use of mouse-tracking vs. the oculomotor methodology and the language of the stimuli (Dutch vs. English), might have contributed to producing the different results. Unfortunately, Bundt and colleagues did not employ a neutral trial baseline so it is not clear whether their effect represents interference, facilitation, or both.

In summary, two studies have reported Stroop effects on response execution; findings that represent a challenge to the currently assumed modularity between response selection and execution. More work is needed to determine what conditions produce Stroop effects on response execution and in which response modalities. Furthermore, it would be interesting for future research to reveal whether semantic and task conflict are registered at this very late stage of selection. For now, this work suggests that even if selection only occurred at the level of response output and not before, it is not always entirely successful, even if the eventual response is correct.

## Locus or loci of selection?

In many early considerations of the Stroop effect, a putative explanation was that interference would not occur unless a name has been generated for the irrelevant dimension; and interference was a form of response conflict due to there being a single response channel (Morton, 1969). Since word reading would more quickly produce a name than color naming it was thought that the word name would be sat in the response buffer before the color name arrived and, thus, would have to be expunged before the correct name could be produced. Thus, Stroop interference was thought to be a consequence of the time it took to process each of the dimensions.

Treisman (1969) questioned why selective attention did not gate the irrelevant word. Treisman concluded that the task of focusing on one dimension whilst excluding the other was impossible, especially when the dimensions are presented simultaneously. Parallel processing of both dimensions would, therefore, occur and thus, response competition could be conceived of as the failure of selective attention to fully focus on the color dimension and gate the input from word processing. Bringing Treisman (1969) and Morton’s (1969) positions together, Dyer (1973) proposed interference results from both a failure in selective attention and a bottleneck at the level of response (at which the word information arrives more quickly). However, the speed-of-processing account has been shown to be unsupported (Glaser & Glaser, 1982; MacLeod & Dunbar, 1988), leaving the failure of attentional selection as the main mechanism leading to Stroop interference.

Whilst it is clear that participants must select a single response in the Stroop task and, thus, that selection occurs at response output, conflict stems from incompatibility between task-relevant and task-irrelevant stimulus features (Egner et al., 2007), and is, thus, stimulus-based conflict. However, even if stimulus incompatibility does make an independent contribution to Stroop interference it might not have an independent selection mechanism; all interference produced at all levels might accumulate and be resolved only later when a single response has to be selected. One way to investigate whether selection occurs at any level other than response output would be to show successful resolution of conflict in the complete absence of response conflict. The 2:1 color-response mapping paradigm is the closest method so far construed that would permit this but as we have explained it is problematic and moreover, it only addresses the distinction between semantic and response conflict.

There are now accounts of the Stroop task which argue that selection occurs both at early and late stages of processing (Altmann & Davidson, 2001; Kornblum & Lee, 1995; Kornblum et al., 1990; Phaf et al., 1990; Sharma & McKenna, 1998; Zhang & Kornblum, 1998; Zhang et al., 1999). For example, in Kornblum and colleagues’ models selection occurs for both SS-conflict and SR-conflict, independently. We have provided evidence for multiple levels of processing contributing to Stroop interference—both stimulus- and response-based contributions. At the level of the stimulus, we have argued that there is good evidence for task conflict. At the level of response, we have argued that the current methods used to dissociate forms of informational conflict including phonological, semantic (stimulus) and response conflict do not permit us to conclude in favor of separate selection mechanisms for each. Moreover, we have discussed evidence that selection at the level of response output is not entirely successful given that response execution effects have been reported.

Another approach would be to show that the different forms of conflict are independently affected by experimental manipulations. Above we alluded to Augustinova and colleagues research showing that semantic conflict is often reported to be preserved in contexts where response conflict is reduced (e.g., Augustinova & Ferrand, 2012). However, we discussed the potential limitations of this approach. Taking another example, in an investigation of the response set effect and non-response set effect, Hasshim and Parris (2018) reported within-subjects experiments in which the trial types (e.g., response set, non-response set, non-color-word neutral) were presented either in separate blocks (pure) or in blocks containing all trial types in a random order (mixed). They observed a decrease in RTs to response set trials when trials were presented in mixed blocks when compared to the RTs to response set trials in pure blocks. These findings demonstrate that presentation format modulates the magnitude of the response set effect, substantially reducing it when trials are presented in mixed blocks. Importantly for present purposes, the non-response set effect was not affected by the manipulation suggesting that the response set and non-response set effects are driven by independent mechanisms. However, Hasshim and Parris’s effect could also be a consequence of the limited effect of presentation format and simply be showing that some conflict is left over—and we do not know which type of conflict it is because the measure was not good enough (see also Hershman et al., 2020; Hershman & Henik, 2019, 2020, showing that conflict can be present but not expressed in the RT data). Future research could further investigate the effect of mixing trial types in blocks on the expression of types of conflict and facilitation in both within- and between-subjects designs.

Kinoshita et al. (2018) argued that semantic Stroop interference can be endogenously controlled evincing independent selection. The authors reported that a high proportion (75%) of non-readable neutral trials (#s) magnified semantic conflict (in the same way this manipulation increases task conflict). This means that a low proportion of non-readable neutral trials leads to reduced semantic conflict. However, since their manipulation was based on the number of non-readable stimuli, Kinoshita et al. (2018) would have also increased task conflict. Neatly, their non-color-related neutral word baseline condition permitted them to show that the semantic component of informational conflict was modulated. Uniquely, in their study they employed both semantic-associative and non-response set trials to measure semantic conflict, perhaps providing converging evidence for a modification of semantic conflict. Problematically, however, they did not include a measure of response conflict in their study so it is not known whether purported indices of response conflict are also affected along with the indices of semantic conflict and thus, their results do not unambiguously represent a modification of semantic conflict. Their study does, however, provide evidence that as task conflict increases, so inevitably does informational conflict because task conflict is an indication that the word is being processed (assuming a sufficient reading age; see Ferrand et al., 2020).

It is our contention that despite attempts to show independence of control of semantic and response conflict, the published evidence so far does not permit a clear conclusion on the matter because the measures themselves are problematic. Future research could combine the semantic distance manipulation (Klopfer, 1996) with a corollary for responses (see, e.g., Chen & Proctor, 2014; Wühr & Heuer, 2018). For example, an effect of the physical (e.g., red in blue, where red is next to blue on a response box vs. red in green when green is further away from the red response key) and conceptual (e.g., red in blue, where the red response is indicated by the key labeled ‘5’ and the blue by a key labeled ‘6’) distance of the response keys has been reported whereby the closer physically or conceptually the response keys, the greater the amount of interference experienced (Chen & Proctor, 2014). Controlling for semantic distance whilst manipulating response distance and vice versa might give an insight into the contributions of semantic and response conflict to Stroop interference by allowing the independent manipulation of both.

In our opinion, methods addressing task conflict, particularly those demonstrating negative facilitation and its control, are evidence for a form of conflict that is independent from response conflict. The evidence for an earlier locus (Hershman & Henik, 2019), distinct developmental trajectory (Ferrand et al., 2020) and independent control (Goldfarb & Henik, 2007; Kalanthroff et al., 2013) support the notion that task conflict has a different locus and selection mechanism to response conflict. Therefore, any model of Stroop performance that does not account for task conflict does not provide a full account of factors contributing to Stroop effects. Only one model currently accounts for task conflict (Kalanthroff et al., 2018) although this model employs the PDP connectionist architecture that falls foul of the word frequency findings noted above.

## Conclusion

Unambiguous evidence that interference (or facilitation) is observed even in the absence of response competition (or convergence) constitutes a necessary prerequisite for moving beyond the historically favored response locus of Stroop effects. In our opinion, task conflict has been shown to be an independent locus for Stroop interference, but phonological, semantic and response conflict (collectively informational conflict) have not been shown to be independent forms of conflict. One could argue that models that incorporate early selection mechanisms are better supported by the evidence, at least in their ability to represent multiple levels of selection that might possibly occur, if not necessarily where that selection occurs since these models do not account for task conflict. Moreover, no extant model can currently predict interference that is observed to occur at the level of response execution and only one model seems able to account for differences in magnitudes of Stroop effects as a function of response modes (Roelofs, 2003).

In short, if the conclusions drawn here are accepted, models of Stroop task performance will have to be modified so they can more effectively account for multiple loci of both Stroop interference and facilitation. This also applies to the implementations of the Stroop task that are currently used in neuropsychological practice (e.g., Strauss et al., 2007) and applied in basic and applied research. As discussed by Ferrand and colleagues (2020), the extra sensitivity of the Stroop test (stemming from the ability to detect and rate each of these components separately) would provide clinical practitioners with invaluable information since the different forms of conflict are possibly detected and resolved by different neural regions. In sum, this review also calls for changes in Stroop research practices in basic, applied and clinical research.

## Data Availability

Not applicable.

## References

[CR1] Algom D, Chajut E (2019). Reclaiming the Stroop effect back from control to input-driven attention and perception. Frontiers in Psychology.

[CR2] Algom D, Chajut E, Lev S (2004). A rational look at the emotional stroop phenomenon: A generic slowdown, not a stroop effect. Journal of Experimental Psychology: General.

[CR3] Algom D, Fitousi D (2016). Half a century of research on Garner interference and the separability–integrality distinction. Psychological Bulletin.

[CR4] Altmann, E. M. & Davidson, D. J. (2001). An integrative approach to Stroop: Combining a language model and a unified cognitive theory. In J. D. Moore & K. Stenning (Eds.), *Proceedings**of**the**23rd**Annual**Conference**of**the**Cognitive**Science**Society* (pp. 21–26). Hillsdale, NJ: Laurence Erlbaum.

[CR5] Augustinova M, Clarys D, Spatola N, Ferrand L (2018). Some further clarifications on age-related differences in Stroop interference. Psychonomic Bulletin & Review.

[CR6] Augustinova M, Ferrand L (2007). Influence de la présentation bicolore des mots sur l'effet Stroop [First letter coloring and the Stroop effect]. Annee Psychologique.

[CR7] Augustinova M, Ferrand L (2012). Suggestion does not de-automatize word reading: Evidence from the semantically based Stroop task. Psychonomic Bulletin & Review.

[CR8] Augustinova M, Ferrand L (2014). Automaticity of word reading evidence from the semantic stroop paradigm. Current Directions in Psychological Science.

[CR9] Augustinova M, Flaudias V, Ferrand L (2010). Single-letter coloring and spatial cuiing do not eliminate or reduce a semantic contribution to the Stroop effect. Psychonomic Bulletin & Review.

[CR10] Augustinova M, Parris BA, Ferrand L (2019). The loci of Stroop interference and facilitation effects with manual and vocal responses. Frontiers in Psychology.

[CR11] Augustinova M, Silvert L, Ferrand L, Llorca PM, Flaudias V (2015). Behavioral and electrophysiological investigation of semantic and response conflict in the Stroop task. Psychonomic Bulletin & Review.

[CR12] Augustinova M, Silvert S, Spatola N, Ferrand L (2018). Further investigation of distinct components of Stroop interference and of their reduction by short response stimulus intervals. Acta Psychologica.

[CR13] Barkley RA (1997). Behavioral inhibition, sustained attention, and executive functions: Constructing a unifying theory of ADHD. Psychological Bulletin.

[CR14] Bench CJ, Frith CD, Grasby PM, Friston KJ, Paulesu E, Frackowiak RSJ, Dolan RJ (1993). Investigations of the functional anatomy of attention using the Stroop test. Neuropsychologia.

[CR15] Berggren N, Derakshan N (2014). Inhibitory deficits in trait anxiety: Increased stimulus-based or response-based interference?. Psychonomic Bulletin & Review.

[CR155] Besner, D., Stolz, J. A., & Boutilier, C. (1997). The stroop effect and the myth of automaticity. *Psychonomic Bulletin & Review*, *4*(2), 221–225. 10.3758/BF03209396 10.3758/BF03209396 21331828

[CR16] Besner D, Stolz JA (1998). Unintentional reading: Can phonological computation be controlled?. Canadian Journal of Experimental Psychology-Revue Canadienne De Psychologie Experimentale.

[CR17] Botvinick MM, Braver TS, Barch DM, Carter CS, Cohen JD (2001). Conflict monitoring and cognitive control. Psychological Review.

[CR18] Braem S, Bugg JM, Schmidt JR, Crump MJ, Weissman DH, Notebaert W, Egner T (2019). Measuring adaptive control in conflict tasks. Trends in Cognitive Sciences..

[CR19] Braver TS (2012). The variable nature of cognitive control: A dual mechanisms framework. Trends in Cognitive Sciences.

[CR20] Brown M, Besner D (2001). On a variant of Stroop’s paradigm: Which cognitions press your buttons?. Memory & Cognition.

[CR21] Brown TL (2011). The relationship between Stroop interference and facilitation effects: Statistical artifacts, baselines, and a reassessment. Journal of Experimental Psychology: Human Perception and Performance.

[CR22] Brown TL, Gore CL, Pearson T (1998). Visual half-field Stroop effects with spatial separation of word and color targets. Brain and Language.

[CR23] Bugg JM, Crump MJC (2012). In support of a distinction between voluntary and stimulus-driven control: A review of the literature on proportion congruent effects. Frontiers in Psychology.

[CR24] Bundt, C., Ruitberg, M. F., Abrahamse, E. L. & Notebaert, W. (2018). Early and late indications of item-specific control in a Stroop mouse tracking study. *PLoS**One,**13*(5), e0197278. 10.1371/journal.pone.0197278 PMC5957332 29771931

[CR25] Burt, J. S. (1994). Identity primes produce facilitation in a colour naming task. *Quarterly**Journal**of**Experimental**Psychology:**Human**Experimental**Psychology,**47*(A), 957–1000.

[CR26] Burt JS (1999). Associative priming in color naming: Interference and facilitation. Memory and Cognition.

[CR27] Burt JS (2002). Why do non-colour words interfere with colour naming?. Journal of Experimental Psychology-Human Perception and Performance.

[CR28] Chen A, Bailey K, Tiernan BN, West R (2011). Neural correlates of stimulus and response interference in a 2–1 mapping Stroop task. International Journal of Psychophysiology.

[CR29] Chen A, Tang D, Chen X (2013). Training reveals the sources of Stroop and Flanker interference effects. PLoS ONE.

[CR30] Chen J, Proctor RW (2014). Conceptual response distance and intervening keys distinguish actions goals in the Stroop Colour-Identification Task. Psychonomic Bulletin and Review.

[CR31] Chen Z, Lei X, Ding C, Li H, Chen A (2013). The neural mechanisms of semantic and response conflicts: An fMRI study of practice-related effects in the Stroop task. NeuroImage.

[CR32] Chuderski A, Smolen T (2016). An integrated utility-based model of conflict evaluation and resolution in the Stroop task. Psychological Review.

[CR33] Cohen JD, Dunbar K, McClelland JL (1990). On the control of automatic processes: A parallel distributed processing account of the Stroop effect. Psychological Review.

[CR34] Coltheart M, Woollams A, Kinoshita S, Perry C (1999). A position-sensitive Stroop effect: Further evidence for a left-to-right component in print-to-speech conversion. Psychonomic Bulletin & Review.

[CR35] Dalrymple-Alford EC (1972). Associative facilitation and interference in the Stroop color-word task. Perception & Psychophysics.

[CR36] Dalrymple-Alford EC, Budayr B (1966). Examination of some aspects of the Stroop color-word test. Perceptual and Motor Skills.

[CR37] De Fockert JW (2013). Beyond perceptual load and dilution: A review of the role of working memory in selective attention. Frontiers in Psychology.

[CR38] De Houwer J (2003). On the role of stimulus-response and stimulus-stimulus compatibility in the Stroop effect. Memory & Cognition.

[CR39] Dennis I, Newstead SE (1981). Is phonological recoding under strategic control?. Memory & Cognition.

[CR156] Dishon-Berkovits, M., & Algom, D. (2000). The Stroop effect: It is not the robust phenomenon that you have thought it to be. *Memory and Cognition*, *28*, 1437–1449. 10.3758/bf03211844 11219971

[CR40] Dyer FN (1973). The Stroop phenomenon and its use in the study of perceptual, cognitive and response processes. Memory & Cognition.

[CR41] Egner T, Delano M, Hirsch J (2007). Separate conflict-specific cognitive control mechanisms in the human brain. NeuroImage.

[CR42] Egner T, Ely S, Grinband J (2010). Going, going, gone: Characterising the time-course of congruency sequence effects. Frontiers in Psychology.

[CR43] Entel O, Tzelgov J (2018). Focussing on task conflict in the Stroop effect. Psychological Research Psychologische Forschung.

[CR44] Entel O, Tzelgov J, Bereby-Meyer Y, Shahar N (2015). Exploring relations between task conflict and informational conflict in the Stroop task. Psychological Research Psychologische Forschung.

[CR45] Ferrand L, Augustinova M (2014). Differential effects of viewing positions on standard versus semantic Stroop interference. Psychonomic Bulletin & Review.

[CR46] Ferrand L, Ducrot S, Chausse P, Maïonchi-Pino N, O’Connor RJ, Parris BA, Perret P, Riggs KJ, Augustinova M (2020). Stroop interference is a composite phenomenon: Evidence from distinct developmental trajectories of its components. Developmental Science.

[CR47] Findlay JM (1982). Global visual processing for saccadic eye movements. Vision Research.

[CR48] Fox LA, Schor RE, Steinman RJ (1971). Semantic gradients and interference in color, spatial direction, and numerosity. Journal of Experimental Psychology.

[CR49] Gazzaniga MS, Ivry R, Mangun GR (2013). Cognitive Neuroscience: The Biology of Mind.

[CR50] Gherhand S, Barry C (1998). Word frequency effects in oral reading are not merely age-of-acquisition effects in disguise. Journal of Experimental Psychology: Learning, Memory and Cognition.

[CR51] Gherhand S, Barry C (1999). Age of acquisition, word frequency, and the role of phonology in the lexical decision task. Memory & Cognition.

[CR52] Glaser WR, Glaser MO (1989). Context effects in stroop-like word and picture processing. Journal of Experimental Psychology: General.

[CR53] Goldfarb L, Henik A (2006). New data analysis of the Stroop matching task calls for a reevaluation of theory. Psychological Science.

[CR54] Goldfarb L, Henik A (2007). Evidence for task conflict in the Stroop effect. Journal of Experimental Psychology: Human Perception and Performance.

[CR55] Gonthier C, Braver TS, Bugg JM (2016). Dissociating proactive and reactive control in the Stroop task. Memory and Cognition.

[CR56] Hasshim N, Bate S, Downes M, Parris BA (2019). Response and semantic Stroop effects in mixed and pure blocks contexts: An ex-Gaussian analysis. Experimental Psychology.

[CR57] Hasshim N, Parris BA (2014). Two-to-one color-response mapping and the presence of semantic conflict in the Stroop task. Frontiers in Psychology.

[CR58] Hasshim N, Parris BA (2015). Assessing stimulus-stimulus (semantic) conflict in the Stroop task using saccadic two-to-one colour response mapping and preresponse pupillary measures. Attention, Perception and Psychophysics.

[CR59] Hasshim N, Parris BA (2018). Trial type mixing substantially reduces the response set effect in the Stroop task. Acta Psychologica.

[CR60] Heathcote A, Popiel SJ, Mewhort DJK (1991). Analysis of response time distributions: An example using the Stroop task. Psychological Bulletin.

[CR61] Henik A, Salo R (2004). Schizophrenia and the stroop effect. Behavioral and Cognitive Neuroscience Reviews.

[CR62] Hershman R, Henik A (2019). Dissociation between reaction time and pupil dilation in the Stroop task. Journal of Experimental Psychology: Learning, Memory and Cognition.

[CR63] Hershman R, Henik A (2020). Pupillometric contributions to deciphering Stroop conflicts. Memory & Cognition.

[CR64] Hershman R, Levin Y, Tzelgov J, Henik A (2020). Neutral stimuli and pupillometric task conflict. Psychological Research Psychologische Forschung.

[CR65] Hock, H. S., & Egeth, H. (1970). Verbal interference with encoding in a perceptual classification task. *Journal**of**Experimental**Psychology,**83*(2, Pt.1), 299–303. 10.1037/h0028512 5480902

[CR66] Hodgson TL, Parris BA, Gregory NJ, Jarvis T (2009). The saccadic Stroop effect: Evidence for involuntary programming of eye movements by linguistic cues. Vision Research.

[CR157] Jackson, J. D., & Balota, D. A. (2013). Age-related changes in attentional selection: Quality of task set or degradation of task set across time? *Psychology and Aging*, *28*(3), 744– 753. 10.1037/a0033159 10.1037/a0033159 PMC3979426 23834491

[CR67] Jiang J, Zhang Q, van Gaal S (2015). Conflict awareness dissociates theta-band neural dynamics of the medial frontal and lateral frontal cortex during trial-by-trial cognitive control. NeuroImage.

[CR158] Jonides, J. & Mack, R. (1984). On the Cost and Benefit of Cost and Benefit. *Psychological Bulletin*, *96*(1), 29–44.

[CR68] Kahneman D, Chajczyk D (1983). Tests of automaticity of reading: Dilution of Stroop effects by color-irrelevant stimuli. Journal of Experimental Psychology: Human Perception and Performance.

[CR159] Kalanthroff, E., Goldfarb, L., Usher, M., & Henik, A. (2013). Stop inter- fering: Stroop task conflict independence from informational conflict and interference. *Quarterly Journal of Experimental Psychology*, *66*, 1356–1367. 10.1080/17470218.2012.741606. 10.1080/17470218.2012.741606 23163896

[CR69] Kalanthroff E, Avnit A, Henik A, Davelaar E, Usher M (2015). Stroop proactive control and task conflict are modulated by concurrent working memory load. Psychonomic Bulletin and Review.

[CR70] Kalanthroff E, Davelaar E, Henik A, Goldfarb L, Usher M (2018). Task conflict and proactive control: A computational theory of the Stroop task. Psychological Review.

[CR72] Kane MJ, Engle RW (2003). Working-memory capacity and the control of attention: The contributions of goal neglect, response competition, and task set to Stroop interference. Journal of Experimental Psychology: General.

[CR73] Kello CT, Plaut DC, MacWhinney B (2000). The task-dependence of staged versus cascaded processing: An empirical and computational study of Stroop interference in speech production. Journal of Experimental Psychology: General.

[CR74] Kim, M.-S. Min, S.-J. Kim, K., & Won, B.-Y. (2006). Concurrent working memory load can reduce distraction: An fMRI study [Abstract]. *Journal**of**Vision,**6*(6):125, 125a, http://journalofvision.org/6/6/125/, doi:10.1167/6.6.125.

[CR75] Kim S-Y, Kim M-S, Chun MM (2005). Concurrent working memory load can reduce distraction. Proceedings of the National Academy of Sciences.

[CR76] Kinoshita S, De Wit B, Norris D (2017). The magic of words reconsidered: Investigating the automaticity of reading color-neutral words in the Stroop task. Journal of Experimental Psychology: Learning Memory and Cognition.

[CR77] Kinoshita, S., Mills, L., & Norris, D. (2018). The semantic stroop effect is controlled by endogenous attention. *Journal**of**Experimental**Psychology:**Learning**Memory**and**Cognition*. DOI: 10.1037/xlm0000552 10.1037/xlm0000552 PMC6711761 29672118

[CR78] Klein GS (1964). Semantic power measured through the interference of words with color-naming. The American Journal of Psychology.

[CR79] Klopfer DS (1996). Stroop interference and color-word similarity. Psychological Science.

[CR80] Kornblum S, Hasbroucq T, Osman A (1990). Dimensional overlap: Cognitive basis for stimulus-response compatibility–a model and taxonomy. Psychological Review.

[CR81] Kornblum S, Lee JW (1995). Stimulus-response compatibility with relevant and irrelevant stimulus dimensions that do and do not overlap with the response. Journal of Experimental Psychology: Human Perception and Performance.

[CR82] La Heij W, van der Heijdan & Schreuder, (1985). Semantic priming and Stroop-like interference in word-naming tasks. Journal of Experimental Psychology: Human Perception and Performance.

[CR83] Laeng B, Torstein L, Brennan T (2005). Reduced Stroop interference for opponent colours may be due to input factors: Evidence from individual differences and a neural network simulation. Journal of Experimental Psychology: Human Perception and Performance.

[CR84] Lakhzoum, D. (2017). Dissociating semantic and response conflicts in the Stroop task: evidence from a response-stimulus interval effect in a two-to-one paradigm. Master’s thesis in partial fulfilment of the requirements for the research Master’s degree in Psychology. Faculty of Psychology, Social Sciences and Education Science Clermont-Ferrand.

[CR85] Lamers MJ, Roelofs A, Rabeling-Keus IM (2010). Selection attention and response set in the Stroop task. Memory & Cognition.

[CR86] Leung H-C, Skudlarski P, Gatenby JC, Peterson BS, Gore JC (2000). An event-related functional MRI study of the Stroop color word interference task. Cerebral Cortex.

[CR87] Levin Y, Tzelgov T (2016). What Klein’s “semantic gradient” does and does not really show: Decomposing Stroop interference into task and informational conflict components. Frontiers in Psychology.

[CR88] Littman R, Keha E, Kalanthroff E (2019). Task conflict and task control: A mini-review. Frontiers in Psychology.

[CR89] Logan GD, Zbrodoff NJ (1979). When it helps to be misled: Facilitative effects of increasing the frequency of conflicting stimuli in a Stroop-like task. Memory and Cognition.

[CR90] Logan GD, Zbrodoff NJ (1998). Stroop-type interference: Congruity effects in colour naming with typewritten responses. Journal of Experimental Psychology-Human Perception and Performance.

[CR91] Lorentz E, McKibben T, Ekstrand C, Gould L, Anton K, Borowsky R (2016). Disentangling genuine semantic Stroop effects in reading from contingency effects: On the need for two neutral baselines. Frontiers in Psychology.

[CR92] Luo CR (1999). Semantic competition as the basis of Stroop interference: Evidence from Color-Word matching tasks. Psychological Science.

[CR93] MacLeod CM (1991). Half a century of research on the Stroop effect: An integrative review. Psychological Bulletin.

[CR94] MacLeod CM (1992). The Stroop task: The" gold standard" of attentional measures. Journal of Experimental Psychology: General.

[CR95] MacLeod CM, Dunbar K (1988). Training and Stroop-like interference: Evidence for a continuum of automaticity. Journal of Experimental Psychology: Learning, Memory, and Cognition.

[CR96] MacLeod CM, MacDonald PA (2000). Interdimensional interference in the Stroop effect: Uncovering the cognitive and neural anatomy of attention. Trends in Cognitive Sciences.

[CR97] Mahon BZ, Garcea FE, Navarrete E (2012). Picture-word interference and the Response-Exclusion Hypothesis: A response to Mulatti and Coltheart. Cortex.

[CR98] Manwell, L. A., Roberts, M. A., & Besner, D. (2004). Single letter colouring and spatial cuing eliminates a semantic contribution to the Stroop effect. *Psychonomic**Bulletin**&**Review,**11*(3), 458–462–817. 10.3758/bf03196595 15376795

[CR99] Marmurek HHC, Proctor C, Javor A (2006). Stroop-like serial position effects in color naming of words and nonwords. Experimental Psychology.

[CR100] Mathews A, MacLeod C (1985). Selective processing of threat cues in anxiety states. Behaviour Research and Therapy.

[CR101] Maurer U, Brem S, Bucher K, Brandeis D (2005). Emerging neurophysiological specialization for letter strings. Journal of Cognitive Neuroscience.

[CR102] McClain L (1983). Effects of response type and set size on Stroop color-word performance. Perceptual & Motor Skills.

[CR103] McSorley E, Haggard P, Walker R (2004). Distractor modulation of saccade trajectories: Spatial separation and symmetry effects. Experimental Brain Research.

[CR104] Melara RD, Algom D (2003). Driven by information: A tectonic theory of Stroop effects. Psychological Review.

[CR160] Melara, R. D., & Mounts, J. R. W. (1993). Selective attention to Stroop dimension: Effects of baseline discriminability, response mode, and practice. *Memory & Cognition*, *21*, 627–645. 10.3758/bf03197195 8412715

[CR105] Monahan JS (2001). Coloring single Stroop elements: Reducing automaticity or slowing color processing?. The Journal of General Psychology.

[CR106] Monsell S, Dolyle MC, Haggard PN (1989). Effects of frequency on visual word recognition tasks: Where are they?. Journal of Experimental Psychology: General.

[CR107] Monsell S, Taylor TJ, Murphy K (2001). Naming the colour of a word: Is it responses or task sets that compete?. Memory & Cognition.

[CR108] Morton J (1969). Categories of interference: Verbal mediation and conflict in card sorting. British Journal of Psychology..

[CR109] Navarrete E, Sessa P, Peressotti F, Dell'Acqua R (2015). The distractor frequency effect in the colour-naming Stroop task: An overt naming event-related potential study. Journal of Cognitive Psychology.

[CR110] Neely, J. H., & Kahan, T. A. (2001). Is semantic activation automatic? A critical re-evaluation. In H.L. Roediger, J.S. Nairne, I. Neath, & A.M. Surprenant (Eds.), *The**Nature**of**Remembering:**Essays**in**Honor**of**Robert**G.**Crowder* (pp. 69–93). Washington, DC: American Psychological Association.

[CR111] Neumann, O. (1980). *Selection**of**information**and**control**of**action.* Unpublished doctoral dissertation, University of Bochum, Bochum, Germany.

[CR112] Parris BA (2014). Task conflict in the Stroop task: When Stroop interference decreases as Stroop facilitation increases in a low task conflict context. Frontiers in Psychology.

[CR161] Parris, B. A., Sharma, D., & Weekes, B. (2007). An Optimal Viewing Position Effect in the Stroop Task When Only One Letter Is the Color Carrier. *Experimental Psychology*, *54*(4), 273–280. 10.1027/1618-3169.54.4.273. 10.1027/1618-3169.54.4.273 17953147

[CR113] Parris BA, Augustinova M, Ferrand L (2019). Editorial: The locus of the Stroop effect. Frontiers in Psychology.

[CR114] Parris BA, Sharma D, Weekes BSH, Momenian M, Augustinova M, Ferrand L (2019). Response modality and the Stroop task: Are there phonological Stroop effects with manual responses?. Experimental Psychology.

[CR115] Parris BA, Wadsley MG, Hasshim N, Benattayallah A, Augustinova M, Ferrand L (2019). An fMRI study of Response and Semantic conflict in the Stroop task. Frontiers in Psychology.

[CR116] Phaf RH, Van Der Heijden AHC, Hudson PTW (1990). SLAM: A connectionist model for attention in visual selection tasks. Cognitive Psychology.

[CR117] Redding GM, Gerjets DA (1977). Stroop effects: Interference and facilitation with verbal and manual responses. Perceptual & Motor Skills.

[CR118] Regan, J. E. (1979). *Automatic**processing*. (Doctoral dissertation, University of California, Berkeley, 1977). Dissertation Abstracts International 39, 1018-B.

[CR119] Repovš G (2004). The mode of response and the Stroop effect: A reaction time analysis. Horizons of Psychology.

[CR120] Risko EF, Schmidt JR, Besner D (2006). Filling a gap in the semantic gradient: Color associates and response set effects in the Stroop task. Psychonomic Bulletin & Review.

[CR121] Roelofs A (2003). Goal-referenced selection of verbal action: Modeling attentional control in the Stroop task. Psychological Review.

[CR122] Roelofs A (2010). Attention and Facilitation: Converging information versus inadvertent reading in Stroop task performance. Journal of Experimental Psychology: Learning, Memory, and Cognition.

[CR123] Scheibe KE, Shaver PR, Carrier SC (1967). Color association values and response interference on variants of the Stroop test. Acta Psychologica.

[CR124] Schmidt JR (2019). Evidence against conflict monitoring and adaptation: An updated review. Psychonomic Bulletin and Review.

[CR125] Schmidt JR, Besner D (2008). The Stroop effect: Why proportion congruent has nothing to do with congruency and everything to do with contingency. Journal of Experimental Psychology: Learning, Memory, and Cognition.

[CR126] Schmidt JR, Cheesman J (2005). Dissociating stimulus-stimulus and response-response effects in the Stroop task. Canadian Journal of Experimental Psychology.

[CR127] Schmidt JR, Hartsuiker RJ, De Houwer J (2018). Interference in Dutch-French bilinguals: Stimulus and response conflict in intra- and interlingual Stroop. Experimental Psychology.

[CR128] Schmidt JR, Notebaert W, Den Bussche V (2015). Is conflict adaptation an illusion?. Frontiers in Psychology.

[CR129] Selimbegovič L, Juneau C, Ferrand L, Spatola N, Augustinova M (2019). The Impact of Exposure to Unrealistically High Beauty standards on inhibitory control. L'année Psychologique/topics in Cognitive Psychology.

[CR130] Seymour PHK (1977). Conceptual encoding and locus of the Stroop effect. Quarterly Journal of Experimental Psychology.

[CR162] Shallice, T. (1988). From Neuropsychology to Mental Structure. Cambridge University Press; Cambridge.

[CR131] Sharma D, McKenna FP (1998). Differential components of the manual and vocal Stroop tasks. Memory & Cognition.

[CR132] Shichel I, Tzelgov J (2018). Modulation of conflicts in the Stroop effect. Acta Psychologica.

[CR133] Singer MH, Lappin JS, Moore LP (1975). The interference of various word parts on colour naming in the Stroop test. Perception & Psychophysics.

[CR134] Spieler DH, Balota DA, Faust ME (1996). Stroop performance in healthy younger and older adults and in individuals with dementia of the Alzheimer's type. Journal of Experimental Psychology: Human Perception and Performance.

[CR135] Steinhauser, M., & Hubner, R. (2009). Distinguishing response conflict and task conflict in the Stroop task: Evidence from ex-Gaussian distribution analysis. *Journal**of**Experimental**Psychology.**Human**Perception**and**Performance,**35*(5), 1398–1412. 10.1037/a0016467 19803645

[CR136] Stirling N (1979). Stroop interference: An input and an output phenomenon. The Quarterly Journal of Experimental Psychology.

[CR137] Strauss E, Sherman E, Spreen O (2007). A compendium of neuropsychological tests: Administration, Norms and Commentary.

[CR138] Stroop JR (1935). Studies of interference in serial verbal reactions. Journal of Experimental Psychology.

[CR139] Sugg MJ, McDonald JE (1994). Time course of inhibition in color-response and word-response versions of the Stroop task. Journal of Experimental Psychology: Human Perception and Performance.

[CR140] Treisman AM (1969). Strategies and models of selective attention. Psychological Review.

[CR141] Tsal Y, Benoni H (2010). Diluting the burden of load: Perceptual load effects are simply dilution effects. Journal of Experimental Psychology: Human Perception and Performance.

[CR142] Turken AU, Swick D (1999). Response selection in the human anterior cingulate cortex. Nature Neuroscience.

[CR143] Tzelgov J, Henik A, Sneg R, Baruch O (1996). Unintentional word reading via the phonological route: The Stroop effect with cross-script homophones. Journal of Experimental Psychology: Learning, Memory and Cognition.

[CR144] Van Veen V, Carter CS (2005). Separating semantic conflict and response conflict in the Stroop task: A functional MRI study. NeuroImage.

[CR145] Van Voorhis BA, Dark VJ (1995). Semantic matching, response mode, and response mapping as contributors to retroactive and proactive priming. Journal of Experimental Psychology: Learning, Memory and Cognition.

[CR146] Virzi RA, Egeth HE (1985). Toward a Translational Model of Stroop Interference. Memory & Cognition.

[CR147] Walker R, Deubel H, Schneider W, Findlay J (1997). Effect of remote distractors on saccade programming: Evidence for an extended fixation zone. Journal of Neurophysiology.

[CR148] Wheeler DD (1977). Locus of interference on the Stroop test. Perceptual and Motor Skills.

[CR149] White D, Risko EF, Besner D (2016). The semantic Stroop effect: An ex-Gaussian analysis. Psychonomic Bulletin & Review.

[CR150] Wühr P, Heuer H (2018). The impact of anatomical and spatial distance between responses on response conflict. Memory and Cognition.

[CR151] Yamamoto I, S. & McLennan, C. T. (2016). A reverse Stroop task with mouse tracking. Frontiers in Psychology.

[CR152] Zahedi, A., Rahman, R. A., Stürmer, B., & Sommer, W. (2019). Common and specific loci of Stroop effects in vocal and manual tasks, revealed by event-related brain potentials and post-hypnotic suggestions. *Journal**of**Experiment**Psychology:**General.* EPub ahead of print: http://dx.doi.org/10.1037/xge0000574 10.1037/xge0000574 30730196

[CR153] Zhang H, Kornblum S (1998). The effects of stimulus–response mapping and irrelevant stimulus–response and stimulus–stimulus overlap in four-choice Stroop tasks with single-carrier stimuli. Journal of Experimental Psychology: Human Perception and Performance.

[CR154] Zhang HH, Zhang J, Kornblum S (1999). A parallel distributed processing model of stimulus–stimulus and stimulus–response compatibility. Cognitive Psychology.

